# Topological Quantum Biomaterials Enable Pyroptosis‐Senescence Coupling for Enhanced Colorectal Cancer Therapy

**DOI:** 10.1002/advs.77968

**Published:** 2026-09-30

**Authors:** Zilu Chen, Kun Mei, Yan Yang, Mengna Xu, Xinyue Dai, Renjun Gu, Fuqiong Zhou, Cheng Wang, Meiqi Chang, Yan Huang, Yu Chen

**Affiliations:** ^1^ Department of Ultrasound Nanjing Hospital of Chinese Medicine Affiliated to Nanjing University of Chinese Medicine Nanjing China; ^2^ Materdicine Lab School of Life Sciences Shanghai University Shanghai China; ^3^ School of Chinese Medicine Nanjing University of Chinese Medicine Nanjing China; ^4^ Central Laboratory Nanjing Hospital of Chinese Medicine Affiliated to Nanjing University of Chinese Medicine Nanjing China; ^5^ Department of Clinical Laboratory Nanjing Hospital of Chinese Medicine Affiliated to Nanjing University of Chinese Medicine Nanjing China; ^6^ Science and Technology Innovation Center Shanghai Municipal Hospital of Traditional Chinese Medicine Shanghai University of Traditional Chinese Medicine Shanghai China

**Keywords:** pyroptosis, quantum biomaterial, therapy‐induced senescence, topological semimetal, ultrasound activation

## Abstract

Inducing pyroptosis in tumor cells has emerged as a promising anticancer strategy. However, under sublethal oxidative stress, a considerable fraction of cancer cells evades pyroptosis and undergoes therapy‐induced senescence, which ultimately drives chronic inflammation and facilitates tumor relapse. Here, we report an ultrasound‐responsive sequential therapeutic strategy that orchestrates the pyroptosis‐senescence axis for improved colorectal cancer therapy. This sequential therapeutic strategy integrates two functional modules, including morphologically engineered Pd_3_Sn topological quantum biomaterials and senolytic agent piperlongumine. Upon ultrasound irradiation, the tailored Pd_3_Sn nanostructures generate reactive oxygen species, which subsequently trigger zDHHC5‐mediated S‐acylation of gasdermin D, thereby initiating pyroptosis in colorectal cancer cells. Benefiting from its facet dependent near Fermi level electronic states and favorable peroxide activation kinetics, Pd_3_Sn achieves substantially improved sonocatalytic performance, allowing for the controlled production of radicals and the accurate triggering of the pyroptosis cascade. Intriguingly, the senescence‐associated secretory phenotype factors released from pyroptotic cells drive the surviving cancer cells into a senescent state. These senescent cells are then selectively eliminated by piperlongumine, effectively abrogating their senescence‐associated pro‐tumorigenic effects. In subcutaneous and liver metastasis models, this nanoplatform significantly suppresses tumor progression, achieving an 83.4% tumor volume inhibition rate and minimal liver metastatic burden. This work opens the application of quantum biomaterials in biomedicine and establishes a new therapeutic paradigm for disease treatment.

## Graphical abstract

This work reports conceptually unprecedented topological quantum biomaterials derived from engineered Pd_3_Sn nanostrips for oncological applications. Their near‐Fermi‐level electronic architecture and superior interfacial charge transport facilitate ultrasound‐amplified redox reactions and efficient ROS generation. Furthermore, this work introduces a novel sequential therapeutic strategy that couples pyroptosis induction with senolytic mediated senescence clearance, thereby overcoming the limitations of conventional cancer therapies that either focus only on tumor cell killing or inadvertently promote therapy induced senescence with pro tumoral consequences.

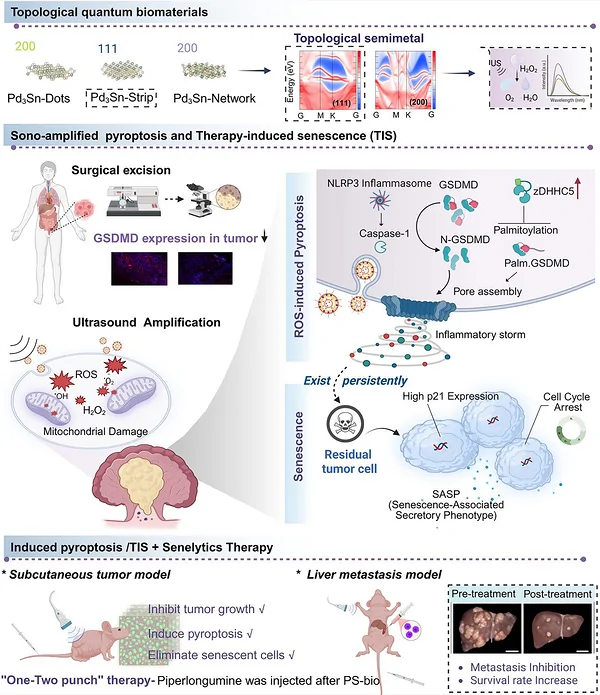

## 1 Introduction

Colorectal cancer (CRC) ranks among the most commonly diagnosed malignancies globally and continues to be a leading cause of cancer‐related mortality [1]. Although advances in surgery, radiochemotherapy, and immunotherapy have contributed to improved patient outcomes, long‐term survival in advanced CRC remains limited [2]. Key barriers to achieving sustained disease control include tumor heterogeneity, therapeutic resistance, and the capacity of cancer cells to evade regulated cell death [3]. A growing body of evidence implicates disrupted redox homeostasis in the facilitation of malignant progression [4, 5]. The oxidative stress‐induced accumulation of reactive oxygen species (ROS) not only constitutes a critical initiator of cell death but also functions as a pivotal determinant of tumor cell fate [3, 6]. Hence, the precise and controlled modulation of intracellular ROS levels to selectively activate specific cell death pathways has emerged as a central objective in contemporary cancer therapy.

Existing studies have confirmed that ROS can effectively induce acute inflammatory responses and trigger strong anti‐tumor immune activity, playing a decisive role in inducing immunogenic programmed cell death [7, 8, 9]. Pyroptosis is an inflammatory form of regulated cell death, which results in the formation of membrane pores, leading to membrane rupture and the subsequent release of intracellular contents [10, 11]. These events can stimulate antitumor immune responses and promote immune‐mediated clearance of cancer cells. Nevertheless, pyroptosis is exquisitely sensitive to both the intensity and duration of upstream stimuli, particularly ROS, underscoring the critical role of the spatiotemporal dynamics of oxidative stress in determining cell fate [12]. During attempts to induce pyroptosis, not all target cells execute the complete lytic cascade. Under sublethal oxidative stress, a proportion of cells escape lysis and remain viable, yet adopt a stable state of cell‐cycle arrest, termed cellular senescence [13, 14, 15].

Cellular senescence can be induced by various stresses and is characterized by long‐lasting proliferation arrest [16, 17]. In the context of anticancer therapy, senescence is often interpreted as a stress‐induced response that curtails tumor progression [18]. Although seemingly beneficial at the outset, senescent cells are not biologically inert [19]. Instead, they secrete a complex repertoire of cytokines, chemokines, proteases, and redox‐related mediators, collectively designated the senescence‐associated secretory phenotype (SASP) [20]. Within the tumor microenvironment, SASP factors perpetuate chronic inflammation, enhance tumor cell survival and immune evasion, induce epithelial‐mesenchymal transition and stem‐like characteristics, and facilitate invasion and metastasis, ultimately establishing a pro‐tumorigenic niche [21]. Therapy‐induced senescence (TIS) has been observed following various treatment regimens, including chemotherapy, radiotherapy, photothermal therapy, and other modalities [22]. Although TIS can transiently suppress tumor growth, the protracted accumulation of senescent cells and their associated SASP may ultimately promote tumor relapse and metastatic dissemination [23, 24]. This has prompted the hypothesis that combining efficient induction of cell death with targeted elimination of TIS cells may constitute a rational strategy for achieving more sustained and durable antitumor responses. Recent studies have begun to translate the distinctive electronic and energy conversion properties of topological materials into biomedical functions. For example, engineered bismuthene nanodiscs have been used to couple topologically enhanced photothermal conversion with thermally regulated gene editing, while multienzyme mimicking bismuthene nanotriggers have been developed to amplify ferroptosis and tumor radiosensitization. These studies illustrate the emerging potential of topological materials in energy conversion, catalytic therapy, and programmable cancer treatment [25, 26]. Recent developments in sonodynamic therapy have largely focused on improving ROS generation and combining US‐activated treatment with phototherapy, chemotherapy, radiotherapy, immunotherapy, or modulation of the tumor microenvironment [27]. Representative studies have further demonstrated that enhancing sonodynamic activity or coupling it with immune regulation can substantially improve tumor control [28, 29]. In parallel, senolytic strategies have been developed to eliminate therapy induced senescent cells and mitigate their persistent tumor promoting effects [13, 30]. However, the sequential combination of US‐activated tumor cell death with subsequent senolytic clearance remains much less explored. In particular, therapeutic strategies that link pyroptosis induction to the subsequent elimination of residual senescent tumor cells remain uncommon.

In this study, we present an ultrasound (US)‐responsive quantum‐coupled nanoplatform that leverages the pyroptosis‐senescence axis against CRC (Scheme 1). The sequential therapeutic strategy integrates Pd_3_Sn topological quantum biomaterials (TQMs), a class of biologically applied Pd_3_Sn nanostructures with theoretically supported topological semimetal electronic characteristics, with the senolytic agent piperlongumine (PL). The topological character of Pd_3_Sn is supported by a nearly quantized Berry phase around the nodal crossing, a near π Zak phase along the [111] direction, and facet dependent surface spectral functions. Compared with conventional catalytic nanomaterials, Pd_3_Sn TQMs offer an additional means of regulating catalytic activity through their electronic structure. Their electronic states near the Fermi level and facet dependent surface environments may facilitate interfacial charge exchange and peroxide activation. Combined with the spatial and temporal control afforded by US irradiation, these features make Pd_3_Sn suitable for locally amplified ROS generation in tumor therapy. Leveraging precise morphological engineering of Pd_3_Sn nanostructures, samples with well‐defined exposed crystallographic facets were procured, and their catalytic behavior was systematically interrogated via both experimental characterization and density functional theory (DFT) calculations. The resulting oxidative stress drives ROS accumulation, activates inflammasome signaling, and increases the expression of palmitoyltransferase zDHHC5 in CRC cells. In turn, zDHHC5 promotes gasdermin D (GSDMD) palmitoylation, enhances its membrane localization and cleavage, and accelerates the generation of N‐GSDMD, ultimately leading to canonical pyroptosis and the emergence of senescent tumor cells. Subsequently, PL is employed to facilitate the clearance of residual senescent cells, thereby establishing a combinatorial therapeutic regimen that integrates pyroptosis induction with senescence removal. The high antitumor efficacy of the pyroptosis‐senescence coupling strategy was validated in both subcutaneous tumor and liver metastasis models. Through the synergistic integration of structural engineering, US‐triggered activation, and cascade redox catalysis, this TQM platform couples ROS modulation with cell‐fate reprogramming, thereby establishing a robust and rationally designed therapeutic strategy for CRC.

**SCHEME 1:**
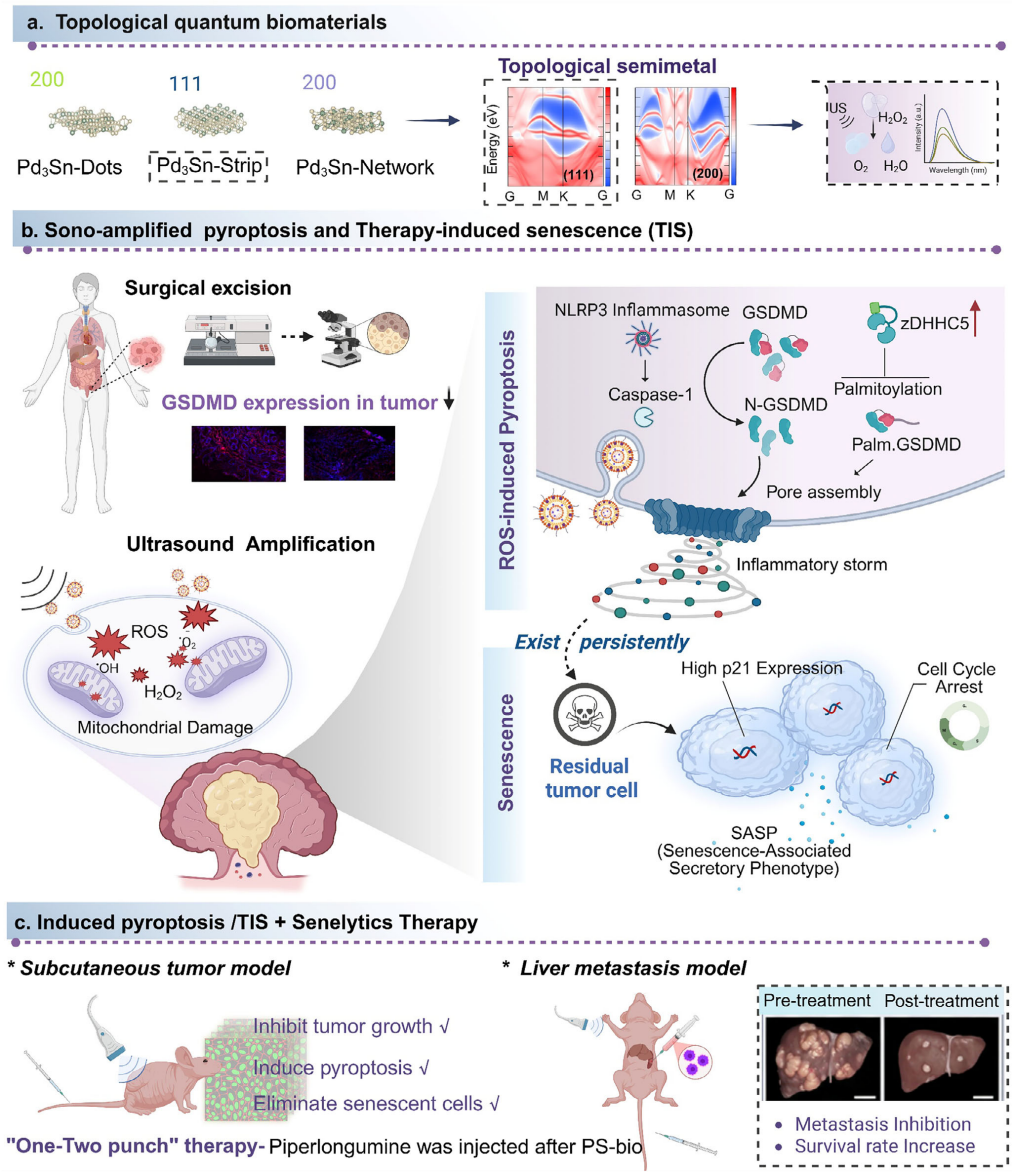
Schematic illustration of US‐responsive topological semimetal platform for CRC therapy. Engineered Pd_3_Sn topological quantum biomaterials and the senolytic agent piperlongumine (PL) were administered separately. Upon US irradiation, Pd_3_Sn generates controlled ROS, triggering zDHHC5‑mediated GSDMD S‑acylation and pyroptosis. Pyroptotic cells release SASP factors, which drive surviving cancer cells into senescence. Subsequently, PL selectively eliminates these senescent cells, blocking their pro‑tumorigenic effects.

## 2 Results and Discussion

### 2.1 Construction and Characterization of Pd_3_Sn TQM and Pd_3_Sn‐Based Nanocomposites

Guided by the idea of tailoring catalytically active facets while preserving the same intermetallic phase, we first developed a morphology‐controlled synthesis of Pd_3_Sn TQM in an oleylamine system (Figure 1a). By modulating the composition of phloroglucinol, glucose, and cetyltrimethylammonium chloride, the growth of Pd_3_Sn could be directed from isotropic nucleation to anisotropic assembly, yielding three distinct morphologies: ultrasmall dots, short strip, and a highly interconnected network (Figure 1b,e,h). The morphology evolution is likely governed by the combined regulation of nucleation and growth kinetics and surface‐selective adsorption during crystal growth. Changes in the reducing and ligand environment can alter the rates of nucleation and atomic deposition, while surfactant‐mediated surface adsorption may further regulate facet growth and favor anisotropic assembly under appropriate conditions. High‐resolution transmission electron microscopy (HRTEM) measurements revealed lattice spacings of 0.198 nm for Pd_3_Sn‐Dots and 0.197 nm for Pd_3_Sn‐Network, which correspond to the (200) crystallographic plane (Figure 1c,i). Pd_3_Sn‐Strip presented a spacing of 0.234 nm corresponding to the (111) plane (Figure 1f). Notwithstanding the distinct facet exposures and morphologies, all three samples exhibited identical powder x‑ray diffraction (XRD) patterns, which refined well to the orthorhombic Pd_3_Sn phase (PDF#37‑0419) with no detectable impurity phases. (Figure 1d,g,j). Tauc analysis of the UV–vis diffuse reflectance spectra yields apparent optical transition energies of 1.92, 1.46, and 1.63 eV for Pd_3_Sn‐Dots, Pd_3_Sn‐Strip, and Pd_3_Sn‐Network, respectively (Figure S1a–c).

**FIGURE 1:**
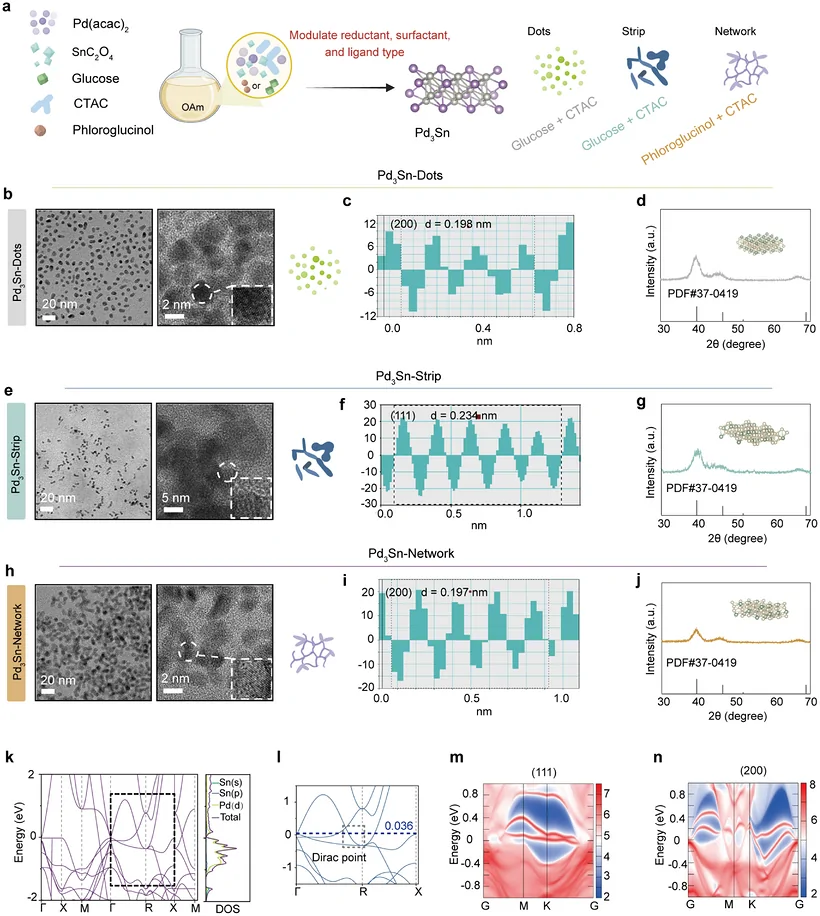
Morphology and crystal structure of Pd_3_Sn topological semimetal nanomaterials with different architectures. (a) Schematic illustration of the colloidal synthesis of Pd_3_Sn TQM nanomaterials. Three distinct nanostructures (Pd_3_Sn‐Dots, Pd_3_Sn‐Strip, and Pd_3_Sn‐Network) are obtained by modulating the combination of reductant, surfactant, and ligand in oleylamine. (b,e,h) TEM and high‐resolution TEM images of Pd_3_Sn‐Dots, Pd_3_Sn‐Strip, and Pd_3_Sn‐Network, respectively. (c,f,i) Line‐intensity profiles corresponding to the lattice fringes in (b,e,h), with the spacings indexed to the (200) and (111) planes of orthorhombic Pd_3_Sn. (d,g,j) Powder x‐ray diffraction patterns of the three as‐synthesized samples. (k) Bulk band structure and total/partial DOS of Pd_3_Sn. (l) Enlarged band structure. (m,n) Surface spectral functions of the Pd_3_Sn (111) and (200) facets along Γ‐M‐K‐Γ.

To link the identical bulk phase with the facet‐dependent catalytic performance, we analyzed the electronic structure of Pd_3_Sn using first principles calculations. The bulk band structure shows semimetallic behavior with band crossings in the vicinity of the Fermi level (*E_F_*), and the total density of States (DOS) displays a clear suppression around *E_F_*, consistent with a semimetal‐like electronic profile. The projected DOS further indicates that the low energy states near *E_F_* are mainly contributed by Pd (d) orbitals, with only minor contributions from Sn states, implying that the electronic channels most relevant to interfacial charge transfer are primarily Pd‐derived (Figure 1k). A magnified band plot resolves a Dirac‐like crossing close to *E_F_*, with an energy offset of approximately 0.036 eV (Figure 1l). The near *E_F_* band crossing is a representative electronic feature of topological semimetal‐like systems, which generally provides high mobility carrier channels and efficient interfacial charge transport. In catalytic and electrochemical processes, this electronic structure can reduce transport losses and facilitate fast electron supply to surface‐active sites, thereby benefiting reaction kinetics [31, 32].

Owing to the non‐negligible spin‐orbit coupling (SOC) arising from the heavy elements Pd and Sn, we additionally performed SOC‐inclusive calculations of the band structure and DOS (Figure S1d,e). These SOC results preserve the overall semimetallic character and maintain the low DOS around *E_F_*, supporting that the near *E_F_* band crossing features are not an artifact of neglecting SOC. In line with the bulk electronic features, surface spectral calculations reveal facet‐dependent surface electronic states, with the (111) plane showing clearer near *E_F_* surface state features than the (200) plane (Figure 1m,n). To establish the topological character independently of visual inspection of the band crossing, a Wannier Hamiltonian was constructed using the Sn s and p orbitals and the Pd s, p, and d orbitals. Without spin orbit coupling, the nodal crossing along the M‐Γ path exhibits a Berry phase of 0.9987π (Figure S1f). Along the [111] direction, 94.5% of the locally gapped projected momentum points show a Zak phase greater than 0.8π (Figure S1g). High precision calculations including spin orbit coupling retain the nearly gapless semimetallic electronic characteristics, with a residual direct gap of 22.5 µeV at the R point (Figure S1d). These bulk topological criteria provide theoretical support for the topological semimetal description of Pd_3_Sn, while the calculated surface spectral functions reveal facet dependent boundary state dispersions (Figure S1h). The present topological classification is supported by theoretical Berry phase, Zak phase, and surface spectral calculations. Direct experimental observation of the surface states by ARPES or STM/STS remains to be investigated. Together, these results indicate that Pd_3_Sn keeps the same bulk phase across different morphologies, while facet engineering modulates the surface electronic environment and the accessibility of near *E_F_* surface states, providing an electronic basis for the observed facet‐sensitive catalytic behavior.

### 2.2 TQM‐Related Electronic Features and ROS Generation

Topological‐semimetal‐related electronic structures can facilitate interfacial charge transfer, which benefits ROS‐generating redox catalysis. To establish the electronic basis underlying the morphology dependent catalytic behavior of Pd_3_Sn, we first summarized the TQM related characteristics of this intermetallic system. As illustrated schematically, the semimetal‐like band feature with near Fermi level electronic states is expected to facilitate interfacial charge transfer and thereby favor ROS‐associated redox catalysis (Figure 2a). To further probe the electrochemical response of three morphologies under tumor‐relevant acidic conditions, linear sweep voltammetry (LSV) measurements were performed in acetate buffer (pH 5.5) with or without H_2_O_2_. All three Pd_3_Sn nanostructures showed enhanced cathodic current responses after the introduction of H_2_O_2_, indicating their ability to participate in interfacial peroxide activation (Figure S2a,b). Notably, when the current density at −0.55 V was extracted for comparison, Pd_3_Sn‐Strip exhibited the largest H_2_O_2_‐dependent current response among the three morphologies, whereas Pd_3_Sn‐Dots and Pd_3_Sn‐Network showed weaker electrochemical activation (Figure 2b). These results support that the strip morphology provides a more favorable near‐surface electronic environment for interfacial charge exchange with peroxide species.

**FIGURE 2:**
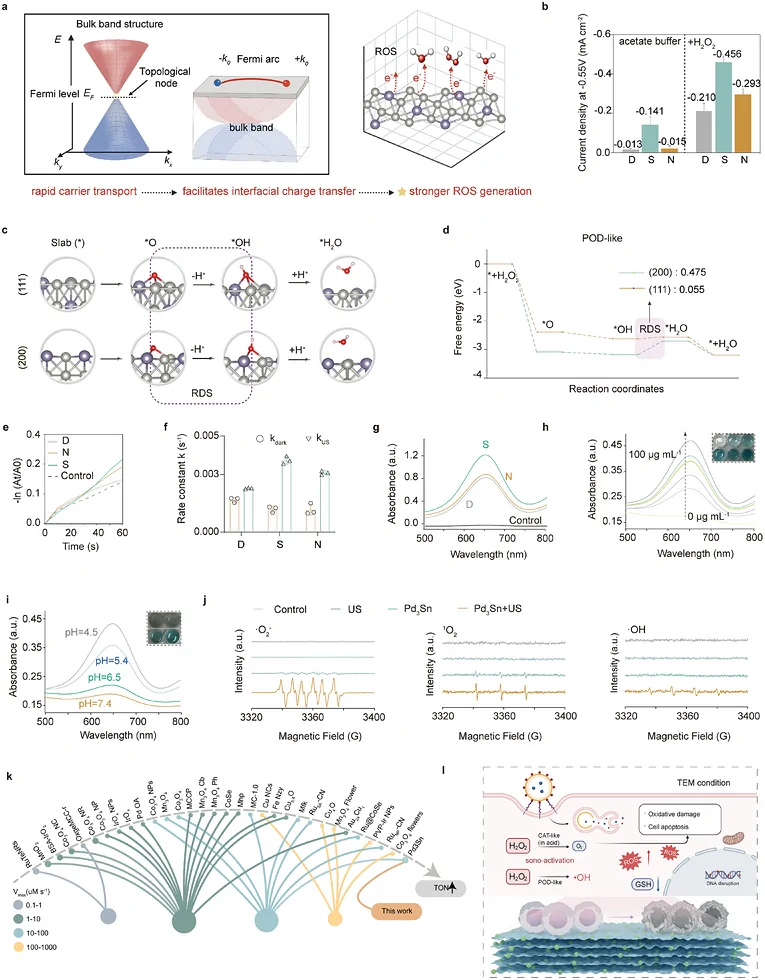
ROS generation characteristics of Pd_3_Sn TQM. (a) Bulk band structure and topological surface states of Pd_3_Sn. Near‐Fermi‐level surface states are proposed to facilitate interfacial charge exchange and enhance ROS generation. (b) LSV measurements of Pd_3_Sn‐Dots (D), Strip (S), and Network (N) in acetate buffer without or with H_2_O_2_ at −0.55 V. (c) Optimized geometries of H_2_O_2_ and reaction intermediates (*O, *OH, *H_2_O) on the (200) and (111) facets of Pd_3_Sn‐Dots, Pd_3_Sn‐Network and Pd_3_Sn‐Strip, respectively. (d) DFT‐calculated free‐energy profiles of the H_2_O_2_ decomposition pathway. (e) Pseudo‐first‐order kinetic plots of DPBF bleaching in the presence of Pd_3_Sn‐Dots, Pd_3_Sn‐Network and Pd_3_Sn‐Strip under US irradiation (1.0 MHz, 1.25 W cm^−2^, 50% duty cycle). (f) Apparent rate constants in the dark (k_dark) and under US (k_US) extracted from panel (e). (g) UV–vis absorption spectra of oxTMB generated in the TMB‐H_2_O_2_ system catalyzed by Pd_3_Sn with different morphologies. (h,i) POD‐like activity of Pd_3_Sn evaluated by the TMB assay. Dependence of oxTMB absorbance on catalyst concentration (h) and pH (i). (j) ESR spectra of DMPO‐•O_2_^−^ adducts, TEMP‐^1^O_2_ adducts and DMPO‐•OH adducts in different groups. (k) Comparison of the maximal reaction velocity (Vmax) and TON of Pd_3_Sn‐Strip with representative reported CAT‐like nanozymes. (l) Schematic illustration of the therapeutic mechanism. US‐triggered Pd_3_Sn generates multiple ROS, leading to oxidative damage and consequent cell death.

To gain molecular level insight into this difference, density functional theory calculations were performed to investigate H_2_O_2_ activation on the representative surfaces of Pd_3_Sn‐Dots, Pd_3_Sn‐Strip, and Pd_3_Sn‐Network. On the examined facets, H_2_O_2_ followed the same elementary reaction sequence, evolving from adsorbed peroxide intermediates to *O, then *OH, and finally *H_2_O (Figure 2c). Analysis of the free energy profiles identified *H_2_O desorption as the rate‐determining step. The calculated barrier was 0.475 eV for the (200) plane and only 0.055 eV for the (111) plane, indicating a much more facile reaction pathway on the strip‐associated facet (Figure 2d). The substantially lower reaction barrier on the (111) facet, together with the stronger H_2_O_2_ dependent electrochemical response of Pd_3_Sn Strip, indicates that its facet dependent electronic structure provides a more favorable environment for peroxide activation. These results support a mechanistic link between the electronic structure of Pd_3_Sn and its enhanced ROS generating activity under US irradiation. The DFT calculations were intended to resolve the intrinsic energetics of facet‐dependent peroxide activation rather than to explicitly model the dynamic acoustic cavitation process. These theoretical and electrochemical results were consistent with the peroxidase‐like catalytic assays. In the 3,3′,5,5′‐tetramethylbenzidine (TMB)‐H_2_O_2_ system, all three Pd_3_Sn nanostructures catalyzed TMB oxidation, as evidenced by the characteristic absorbance around 652 nm, with Pd_3_Sn‐Strip showing the strongest activity among the three morphologies (Figure S2c–e).

To evaluate the overall US‐induced oxidative activity of the Pd_3_Sn nanostructures, 1,3‐diphenylisobenzofuran (DPBF) was used as a probe. The DPBF oxidation process followed pseudo first order kinetics, and the apparent rate constants obtained under US irradiation and dark conditions were defined as *k_US_* and *k_dark_*, respectively (Figure 2e). The *k_US_* values were markedly higher than the corresponding *k_dark_* values across all morphologies, with Pd_3_Sn‐Strip exhibiting the highest enhancement (Figure 2f and Figure S2f). These findings support that both tailored facet exposure and external acoustic excitation synergistically enhance ROS generation activity. Moreover, the absorbance of oxTMB shows a dose‐dependent increase with concentration up to 100 µg mL^−1^ (Figure S2g). Furthermore, variation in the concentrations of H_2_O_2_ and TMB substrates reveals characteristic saturating Michaelis‐Menten kinetics (Figure S2h,i). These results collectively indicate that the strip architecture retains robust catalytic efficiency across physiologically relevant substrate concentration ranges.

Further evaluation of the oxidase‐like activity was performed using a TMB chromogenic system in the absence of exogenous H_2_O_2_. Among the tested samples, Pd_3_Sn‐Strip demonstrated the highest catalytic activity (Figure 2g). The catalytic signal intensified with increasing Pd_3_Sn concentration, reaching saturation at 100 µg mL^−1^ (Figure 2h). Furthermore, the oxidase‐like activity exhibited strong pH dependence, with a maximum under mildly acidic conditions (pH 5.4–5.8), which corresponds to the acidic pH of the tumor microenvironment (Figure 2i). This pH preference may help confine catalytic ROS production to acidic tumor regions and thus improve the therapeutic selectivity of nanocatalyst [33, 34]. Simultaneously, oxygen evolution resulting from H_2_O_2_ decomposition was monitored with a dissolved oxygen meter. Pd_3_Sn‐Strip demonstrated rapid O_2_ generation. Kinetic analysis yielded V_max_ and turnover number (TON) values that match or surpass those of many reported catalase‐like nanosystems (Figure 2k and Figure S2j), underscoring its high efficiency in the catalase‐like conversion of H_2_O_2_ into O_2_. To determine whether the surface modification affects the catalytic activity of Pd_3_Sn‐Strip, we directly compared Pd_3_Sn‐Strip and PS‐bio under identical assay conditions. PS‐bio retains a comparable ability to promote DPBF degradation, indicating that its overall ROS related activity was largely preserved after silica and biotin‐PEG modification (Figure S2k). In the TMB oxidation assay, PS‐bio shows a slightly lower absorbance than Pd_3_Sn‐Strip, suggesting a modest reduction in peroxidase‐like activity after surface coating (Figure S2l). Overall, these results indicate that the surface modification does not markedly compromise the catalytic function of Pd_3_Sn‐Strip, although partial shielding of accessible surface sites may slightly affect its activity.

Electron spin resonance (ESR) spectroscopy was employed to determine the identity of ROS generated under US irradiation. In the presence of both Pd_3_Sn‐Strip and US, the characteristic signals corresponding to DMPO‐•O_2_^−^, TEMP‐^1^O_2_ and DMPO‐•OH adducts are significantly intensified compared to the control and US‐only groups, demonstrating the simultaneous generation of •O_2_^−^, ^1^O_2_ and •OH. Taken together, the combined theoretical and experimental results identify the Pd_3_Sn‐Strip as acoustically activatable TQM with strong catalytic activities and broad‐spectrum ROS production, which provides the mechanistic basis for US‐triggered oxidative stress amplification and subsequent tumor cell killing (Figure 2j).

Considering both structural accessibility and catalytic performance, Pd_3_Sn‐Strip was selected as the core scaffold for further surface engineering. As illustrated in Figure S3a, Pd_3_Sn‐Strip was first coated with a thin silica layer via tetraethyl orthosilicate hydrolysis in cyclohexane, and the silica surface was then modified with 3‐aminopropyltriethoxysilane to introduce amino groups. These amino moieties served as anchoring sites for the covalent conjugation of biotin‐PEG‐NHS, yielding the targeted biotin‐functionalized nanosystem, denoted as Pd_3_Sn‐biotin (PS‐bio). Biotin was selected as a small molecule targeting ligand to enhance the cellular uptake of PS‐bio in tumor cells. Previous studies have shown that biotin modification can promote cellular uptake and tumor delivery of nanocarriers, while biotin modified silica nanoparticles exhibit enhanced accumulation in HCT116 colorectal cancer cells [35, 36]. Scanning transmission electron microscopy‐dark field imaging and corresponding elemental mapping reveal a strong spatial correlation among Pd, Sn, Si, and O, confirming a well‐defined core–shell architecture with a uniform coating on the strip (Figure S3b). Dynamic light scattering further reveals that the hydrodynamic diameter increases from ∼189.5 nm for bare Pd_3_Sn‐Strip to ∼256.8 nm after full surface modification, while the zeta potential shifts from nearly neutral (+1.624 mV) to slightly negative (−2.614 mV) (Figure S3c,d), consistent with the formation of a silica/amino/biotin‐PEG shell that improves colloidal stability and offers abundant functional groups for biological interfacing. The colloidal stability of PS‐bio was further evaluated in PBS and culture medium containing 10% fetal bovine serum at 37°C. Over 48 h, the hydrodynamic diameter remained within 183.5–200.6 nm in PBS and 230.8–256.6 nm in the serum medium, with corresponding polydispersity index (PDI) values of 0.1448–0.1932 and 0.21–0.27, respectively (Figure S4a,b). The absence of abrupt changes in either parameter supports the colloidal stability of PS‐bio under the tested conditions.

The chemical evolution during this stepwise modification was corroborated by Fourier transform infrared spectroscopy (FT‐IR) and x‐ray photoelectron spectroscopy (XPS) analyses. FT‐IR spectra show the appearance of Si‐O‐Si stretching bands after silica coating, followed by new amide‐related vibrations after biotin‐PEG conjugation (Figure S3e), validating the successful construction of the hybrid organic‐inorganic shell. High‐resolution XPS spectra disclose mixed‐valence states of tin (Sn^0^/Sn^2+^/Sn^4+^) and palladium (Pd^0^/Pd^2+^), which are beneficial for redox cycling and catalytic activity (Figure S3f). The survey spectrum confirms the coexistence of Pd, Sn, Si, O, C and N elements in PS‐bio (Figure S3g), while the Si‐O‐Si vibration in the FT‐IR spectrum and Si/O elemental mapping further support the formation of the silica‐containing surface layer.

### 2.3 In Vitro Anti‐Tumor Activity of PS‐bio

The cytotoxic effect of PS‐bio on CRC cells was further examined in the presence or absence of US irradiation. Dose response analyses reveal that PS‐bio alone reduced cell viability in a concentration dependent manner, with viability decreasing to 37.9% ± 2.1% at 200 µg/mL and 17.2% ± 0.3% at 400 µg/mL (Figure S5a). In contrast, the combination of PS‐bio with US triggered a more pronounced reduction in cell viability, especially at relatively lower concentrations, with viability decreasing to 35.0% ± 3.8% at 100 µg/mL and further to 12.0% ± 2.7% at 400 µg/mL (Figure S5b). Treatment with PS‐bio alone resulted in a minimal population of PI positive cells. In contrast, the PS‐bio + US group exhibits extensive PI uptake and a significant decrease in Calcein‐AM‐positive viable cells (Figure S5c), confirming a pronounced US‐triggered cytotoxic effect at the single‐cell level. To further assess the cytocompatibility of PS‐bio in nonmalignant colonic cells, cell viability was evaluated in normal human colonic epithelial NCM460 cells. NCM460 cells show relatively preserved viability at low to moderate PS‐bio concentrations, with a gradual reduction observed only as the concentration increased (Figure S5d). These results indicate acceptable cytocompatibility of PS‐bio toward normal colonic epithelial cells under the tested conditions.

Flow cytometry analysis using the general ROS probe 2',7'‐Dichlorodihydrofluorescein diacetate (DCFH‐DA) revealed that the PS‐bio + US group exhibited a pronounced shift toward higher fluorescence intensity relative to the control, US‐only, and PS‐bio‐only groups (Figure 3a), confirming that the combination treatment induces the greatest intracellular ROS level. We then probed whether this toxicity was associated with oxidative stress and mitochondrial damage. Co‐staining results with MitoSOX and MitoTracker Green reveal a strong accumulation of mitochondrial superoxide in the PS‐bio + US group, where intense red signals colocalized with mitochondria (Figure 3b). Mitochondrial membrane potential results show predominantly red J‐aggregates in the control and US groups, indicative of polarized mitochondria, whereas PS‐bio + US group causes a clear shift toward green monomer fluorescence and a flattened profile (Figure 3c and Figure S5e), indicating severe mitochondrial depolarization. Given that mitochondrial ROS and loss of membrane potential are key upstream events in multiple programmed cell‐death pathways [37, 38], these data suggest that PS‐bio in combination with US induces mitochondrial oxidative stress and mitochondrial membrane depolarization rather than causing non‐specific necrosis.

**FIGURE 3:**
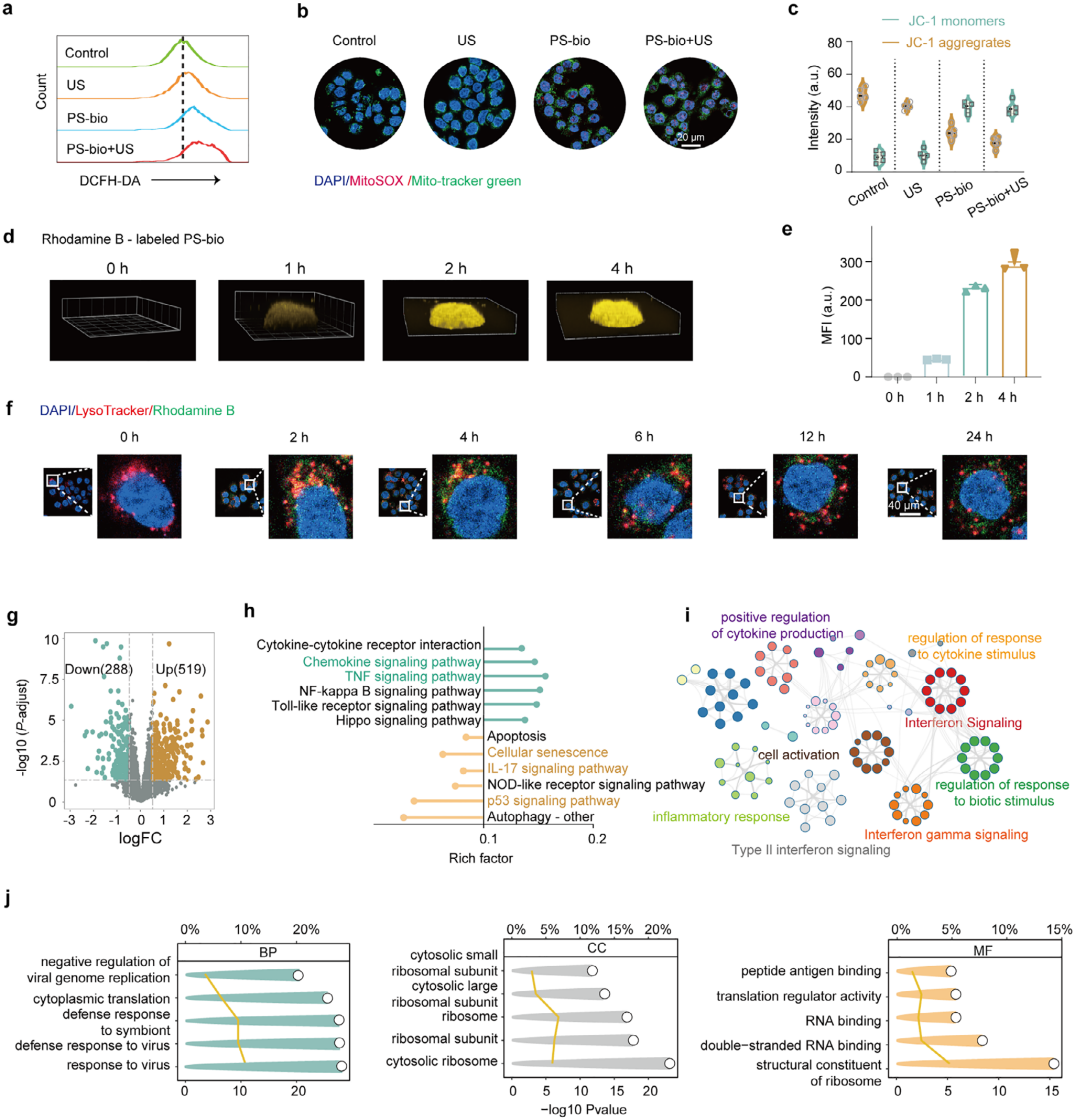
In vitro cellular assays. (a) Overall intracellular ROS levels analyzed by flow cytometry using DCFH‐DA. (b) Confocal images of mitochondrial ROS and morphology visualized by MitoSOX Red (mitochondrial superoxide, red) and MitoTracker Green (mitochondria, green). (c) Mitochondrial membrane potential (ΔΨm) assessed by JC‐1 staining. (d,e) Time‐dependent uptake of PS‐bio in 3D tumor spheroids. Representative confocal z‐stack images of spheroids incubated with fluorescently labeled PS‐bio at indicated time points (d) and corresponding mean fluorescence intensity quantitation within spheroids (e). (f) Time‐dependent cellular uptake and lysosomal escape of PS‐bio during 0–24 h incubation. Nuclei were stained with DAPI (blue), lysosomes were labeled with LysoTracker Green (green), and PS‐bio was conjugated to a red fluorophore. Enlarged views of representative cells are shown in the bottom panels. (g) Volcano plot of DEGs between control and PS‐bio + US group (n = 3). (h) KEGG pathway enrichment of DEGs. (i) GO enrichment analysis between control and PS‐bio group. (j) GO enrichment analysis for BP, CC, and MF categories.

To better simulate the structure of solid tumors, we detected the behavior of PS‐bio in three‐dimensional tumor spheres. Confocal Z‐stack images reveal that PS‐bio fluorescence penetrated from the spheroid periphery into deeper regions over time, accompanied by a continuous increase in mean fluorescence intensity (Figure 3d,e). To distinguish inward distribution from surface accumulation, radial fluorescence profiles were further analyzed from the spheroid surface toward the center. Although fluorescence intensity decreases with increasing depth at all time points, the decline becomes progressively less pronounced with prolonged incubation. The 4 h group retains the highest normalized fluorescence intensity in deeper regions, followed by the 2 h and 1 h groups (Figure S5f), supporting a time dependent extension of PS‐bio toward the spheroid interior. These results indicate that PS‐bio is effectively internalized and achieves a spatiotemporal distribution within dense cellular aggregates, indicating its capacity to penetrate tumor‐like tissue architectures. To elucidate the subsequent intracellular trafficking mechanism by which PS‐bio reaches its catalytic sites, its endo‐lysosomal transport pathway was subsequently investigated. Time‐lapse confocal images reveal that PS‐bio initially localized as discrete puncta, exhibiting strong colocalization with lysosomal markers in the perinuclear region. With prolonged incubation, these puncta demonstrated progressive dissociation from the lysosomal compartment and subsequently diffused throughout the cytosol (Figure 3f). Quantitative colocalization analysis further shows a progressive decrease in Pearson's correlation coefficient between PS‐bio and LysoTracker signals from 2 to 24 h (Figure S5g), supporting a time dependent reduction in lysosomal retention. This pattern is characteristic of efficient lysosomal escape, which allows PS‐bio to avoid lysosomal sequestration and redistribute from lysosomal compartments into the cytosol [39]. Efficient escape from acidic vesicles is considered an important prerequisite for many nanocatalytic systems that rely on intracellular cascade reactions [40]. Collectively, PS‐bio exhibits low off‐target toxicity but, upon US activation, induces pronounced mitochondrial and global ROS stress, benefits from lysosomal escape, and efficiently infiltrates 3D tumor spheroids. These features together underpin its promising in vitro antitumor activity and support its further evaluation for ROS‐mediated cancer therapy.

To obtain a systems‐level understanding of the cellular response to PS‐bio + US treatment, RNA sequencing was performed on HT‐29 cells in the Control and PS‐bio + US groups. Differentially expressed genes (DEGs) were identified using thresholds of |log2 fold change| > 1 and adjusted *p* < 0.05. A total of 519 genes were significantly upregulated and 288 genes were downregulated following PS‐bio+ US treatment (Figure 3g). KEGG analysis of the upregulated DEGs reveals significant enrichment in pathways related to cellular senescence, chemokine signaling, cytokine receptor interaction, IL‐17 signaling, TNF signaling, NF‐κB signaling, and other inflammatory pathways (Figure 3h). These transcriptional changes indicate a broad inflammatory and cellular stress response following treatment. GO analysis further shows that the upregulated genes are mainly associated with inflammatory response, cellular activation, peptide antigen binding, ribosomal processes, and translation, covering biological process, cellular component, and molecular function categories (Figure 3i,j). Together, the transcriptomic results characterize the broader inflammatory and stress responses accompanying treatment, whereas the molecular and functional evidence for pyroptosis is provided by the subsequent protein, morphological, and membrane damage analyses.

### 2.4 Pyroptosis Induction and TIS

Pyroptosis, a lytic and inflammatory mode of programmed cell death, is characterized by cellular swelling, plasma membrane rupture, and the release of intracellular contents [41]. To assess the clinical relevance of targeting this pathway, we first evaluated the expression of GSDMD, the key pore‐forming executioner protein of pyroptosis, in human CRC specimens. Immunofluorescence staining results reveal a significantly reduced GSDMD expression in tumor tissues compared to paired normal colon samples (Figure S6a,b). This finding indicates an impaired pyroptotic execution program in clinical tumors and underscores the translational potential of restoring GSDMD‐dependent pyroptosis.

Bright‐field (Figure S7a) and scanning electron microscopy (SEM) images reveal that cells in the control and US‐only groups maintained a smooth surface and intact plasma membrane (Figure 4a). Treatment with PS‐bio alone induced only mild surface roughening. In contrast, the combination of PS‐bio and US resulted in marked cellular swelling and the emergence of numerous bubble‐like protrusions on the plasma membrane, along with severely disrupted and perforated surfaces. This morphology differs from apoptosis, which is typically characterized by cell shrinkage, membrane blebbing, and apoptotic body formation while membrane integrity is initially preserved. Necrosis or necroptosis may also involve cellular swelling and membrane rupture, but generally lacks the prominent balloon‐like membrane protrusions observed here. Therefore, the imaging results are morphologically consistent with pyroptosis and can be distinguished from the typical features of apoptosis and nonspecific necrotic death. To elucidate the underlying molecular signaling, we next investigated key components of the inflammasome‐pyroptosis cascade. Western blot analysis demonstrated that the combination of PS‐bio and US substantially upregulated the protein expression levels of NLRP3, cleaved caspase‐1, and the N‐terminal fragment of gasdermin D (GSDMD‐N). In contrast, negligible changes in these protein levels were observed in the control, US‐only, and PS‐bio‐only groups (Figure 4b). Quantification results confirm the highest ratios of GSDMD‐N to full‐length GSDMD and of cleaved caspase‐1 to Vinculin in the combination group, indicative of a robust activation of the canonical NLRP3‐caspase‐1‐GSDMD signaling axis (Figure S7b). Functionally, PS‐bio in combination with US induced a sharp depletion of intracellular adenosine triphosphate (ATP) (Figure S7c) and significantly elevated the release of lactate dehydrogenase (LDH) into the supernatant (Figure S7d), indicating extensive membrane damage and lytic cell death. Concomitant with pyroptotic cell death, PS‐bio plus US treatment amplified inflammatory signaling. Enzyme‐linked immunosorbent assay analyses reveal markedly higher secretion levels of the key inflammasome‐associated cytokines IL‐1β and IL‐18 in the PS‐bio plus US group relative to all other treatment groups (Figure S7e).

**FIGURE 4:**
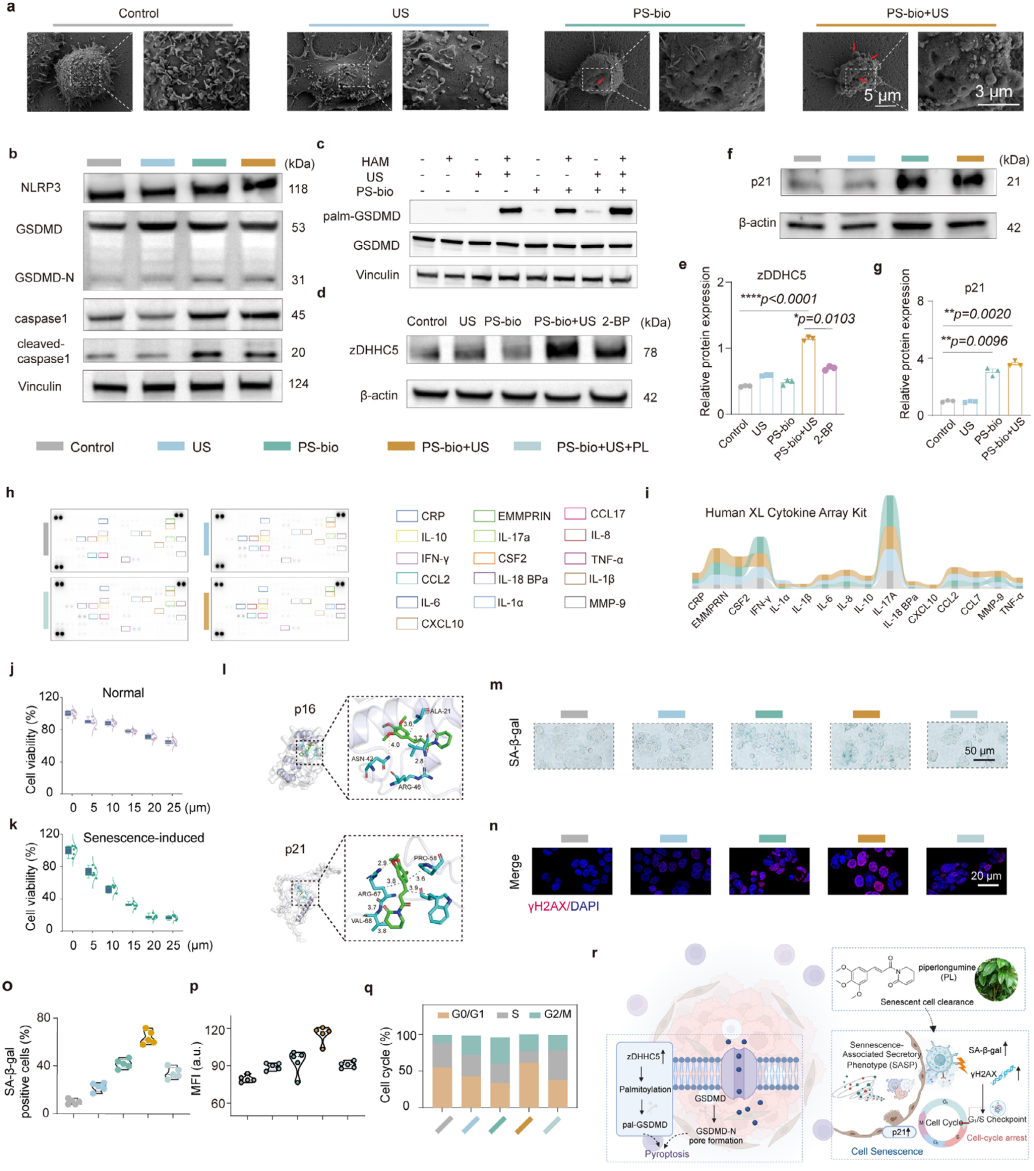
Pyroptosis induction and TIS in vitro. (a) Representative SEM images of cells in the indicated experimental groups. (b) Representative western blot of NLRP3, GSDMD (full‐length and N‐terminal fragment), caspase‐1, and cleaved caspase‐1 across experimental groups. (c) ABE assay showing palmitoylated GSDMD (palm‐GSDMD) levels under different treatment conditions in the presence or absence of US, PS‐bio, and HAM. (d,e) Western blot analysis of zDHHC5 expression and densitometric quantification normalized to β‐actin in Control, US, PS‐bio, PS‐bio + US, and 2‐BP‐treated groups. (f,g) Western blot analysis of p21 expression and densitometric quantification normalized to β‐actin. (h) Cytokine array analysis of secreted factors, highlighting key SASP‐associated cytokines and chemokines. (i) Semi‐quantitative analysis of selected cytokines/chemokines from the human XL cytokine array. (j,k) Cell viability measured by CCK‐8 assay in normal and senescence‐induced HT‐29 cells after PL treatment. (l) Docking simulations showing PL interactions with key senescence regulators p16 and p21. (m,o) Representative SA‐β‐gal staining images and quantitative analysis of SA‐β‐gal‐positive cells. (n,p) Representative immunofluorescence images of γH2AX staining and the corresponding quantitative analysis. (q) Quantitative representation of G0/G1, S, and G2 phase populations based on flow cytometry data. (r) Simplified schematic of pyroptosis and TIS, including GSDMD palmitoylation, SASP activation, DNA damage, cell‐cycle arrest, and senescent cell clearance by PL. Data are presented as mean ± SD. Data in (e), (g), (o), and (p) were analyzed using one‐way ANOVA followed by Tukey's multiple‐comparison test. Cell‐viability data in (j) and (k) were analyzed using one‐way ANOVA followed by Dunnett's multiple‐comparison test. Cell‐cycle distributions in (q) were analyzed using two‐way ANOVA followed by Šídák's multiple‐comparison test. The specific groups included in each comparison are indicated by connecting lines. *p < 0.05, **p < 0.01, ***p < 0.001, and ****p < 0.0001; ns, not significant.

Although mitochondrial ROS accumulation and membrane depolarization are commonly associated with intrinsic apoptosis, these events can also promote NLRP3 inflammasome activation. In the present system, the subsequent cellular swelling and membrane perforation, together with the activation of NLRP3 and caspase‐1, the generation and palmitoylation of GSDMD‐N, ATP depletion, LDH release, and increased secretion of IL‐1β and IL‐18, indicate that mitochondrial oxidative stress is primarily coupled to the NLRP3‐caspase‐1‐GSDMD pyroptotic pathway. Thus, mitochondrial injury serves as an upstream oxidative trigger, whereas the downstream molecular and morphological features identify pyroptosis as the predominant cell death response.

Given the established role of S‐acylation in regulating GSDMD activation, we further investigated GSDMD palmitoylation using an acyl‐biotin exchange (ABE) assay [42]. A distinct palmitoylated‐GSDMD band was detected exclusively in the +hydroxylamine (HAM) samples derived from PS‐bio + US‐treated cells, whereas the ‐HAM controls and other treatment groups show minimal or undetectable signal (Figure 4c). The corresponding schematic (Figure S8) outlines the key steps of the ABE workflow, wherein thioester bonds are selectively cleaved by hydroxylamine to expose acylated cysteine residues for subsequent biotinylation and pulldown. In alignment with this finding, the broad‐spectrum palmitoylation inhibitor 2‐bromopalmitate (2‐BP) attenuates the PS‐bio + US‐induced upregulation of the palmitoyl acyltransferase zDHHC5 [43] (Figure 4d,e). Notwithstanding the incomplete specificity of 2‐BP, this pharmacological perturbation, supports a mechanistic framework in which PS‐bio plus US promotes zDHHC5‐dependent S‐acylation of GSDMD and its subsequent membrane localization, thereby enabling pore formation and the induction of pyroptotic cell death. To further verify the role of zDHHC5, three independent siRNAs targeting zDHHC5 were evaluated. Both Western blotting and RT‐qPCR confirm reduced zDHHC5 expression after siRNA transfection, with the selected siRNA producing a marked knockdown at both the protein and mRNA levels (Figure S9a,b). Genetic depletion of zDHHC5 also reduces the increase in GSDMD palmitoylation induced by PS‐bio + US, as determined by the ABE assay (Figure S9c). These results provide additional genetic evidence that zDHHC5 contributes to GSDMD palmitoylation following PS‐bio + US treatment.

In parallel, we assessed whether the surviving cell population exhibited features of TIS. Western blot analysis reveals a significant upregulation of the cyclin‐dependent kinase inhibitor p21 following PS‐bio treatment, which was further elevated in the PS‐bio + US group (Figure 4f,g), consistent with a senescence‐associated growth arrest. Furthermore, cytokine array results demonstrate a broad‐spectrum upregulation of multiple inflammatory cytokines and chemokines specifically after PS‐bio + US treatment (Figure 4h). The overall secretory trend is illustrated quantitatively in the corresponding profile (Figure 4i), indicating a pronounced inflammatory response after PS‐bio + US treatment. Among the detected factors, IL‐6, IL‐8, IL‐1β, and TNF‐α show increased signals, suggesting that multiple inflammatory mediators may contribute to senescence in neighboring cells. Consistent with this possibility, conditioned medium collected from PS‐bio + US‐treated cells significantly increases SA‐β‐gal positivity in untreated HT‐29 cells compared with control conditioned medium, supporting a paracrine effect of soluble factors released after treatment (Figure S10a,b). However, the cytokine array provides only semi‐quantitative information and cannot identify which factor plays the dominant role. Further studies using specific cytokine blockade are needed to determine the contribution of individual factors.

### 2.5 Senescence Clearance Effect

Given the finding that residual cells undergo TIS following PS‐bio plus US treatment, we subsequently investigated the effect of combining the natural senolytic agent PL to achieve selective clearance of this senescent cell population [44, 45, 46]. CCK‐8 assays reveal that senescence HT‐29 cells exhibit a dose‐dependent decrease in viability upon PL treatment, whereas normal cells remain largely unaffected at the same concentrations (Figure 4j,k), confirming the selective cytotoxicity of PL toward senescent cells. Docking simulations further support the mechanistic basis for this senolytic effect, showing that PL forms multiple hydrogen‐bond and π‐π interactions within the predicted binding pockets of key senescence regulators p16 and p21 (Figure 4l).

Consistently, SA‐β‐Gal staining results confirm that PL treatment substantially reduced the proportion of β‐galactosidase positive senescent cells in the PS‐bio + US group (Figure 4m,o). PS‐bio + US treatment induced a marked accumulation of senescent cells, with SA‐β‐Gal positive cells reaching 64.0% ± 5.8%, whereas this proportion decreased to 34.4% ± 4.7% after PL treatment. Immunofluorescence staining results for γH2AX demonstrate that cells treated with PS‐bio + US displayed pronounced DNA damage foci [47], while PL treatment markedly reduced γH2AX fluorescence intensity, as reflected by the decrease in MFI from 115.2 ± 6.9 to 90.6 ± 2.6 a.u. (Figure 4n,p). Correspondingly, flow cytometric analyses revealed that PS‐bio + US treatment induced a pronounced G0/G1 phase arrest, a characteristic cell cycle alteration commonly observed in senescent cells [48], whereas PL partially reversed this effect, reducing the G0/G1 population from 61.0% to 38.3% and increasing the S phase fraction from 16.7% to 37.4% (Figure 4q and Figure S11). Taken together, these results demonstrate that PL functions as an effective senolytic agent, selectively eliminating therapy‐induced senescent cells, alleviating DNA damage, and partially restoring cell cycle progression, thereby further enhancing the therapeutic efficacy of US‐activated PS‐bio in vitro (Figure 4r).

### 2.6 In Vivo Antitumor Effect

Building on the potent in vitro pyroptosis‐inducing activity of PS‐bio upon US activation, we next assessed its in vivo therapeutic efficacy and explored whether senolytic intervention could potentiate the antitumor effect. A subcutaneous colorectal carcinoma model was established in BALB/c nude mice by injection of CT26 cells. Tumor‐bearing mice were then randomly allocated into six treatment groups (n = 5 per group): G1 (Control), G2 (US only), G3 (PS‐bio only), G4 (PL only), G5 (PS‐bio + US), and G6 (PS‐bio + US + PL). PS‐bio was administered intravenously on days 1, 3, 5, and 7. For the senolytic combination group (G6), PL was subsequently administered on days 10, according to the treatment schedule outlined in the schematic (Figure 5a). This delayed schedule was intended to allow residual tumor cells to establish a senescence‐associated phenotype before senolytic intervention. Earlier administration may precede the establishment of TIS, whereas excessive delay may prolong the persistence of senescent cells. Individual and averaged tumor growth curves reveal rapid tumor progression in the control and US‐only groups. Treatment with PS‐bio alone or PL alone results in only a modest retardation of tumor growth (Figure 5c and Figure S12a). In contrast, treatment with the combination of PS‐bio and US significantly inhibited tumor growth over the 14‑day observation period, reducing the endpoint tumor volume to 664.4 ± 115.2 mm^3^, which corresponded to a tumor volume inhibition rate of 50.2%. The strongest suppression was observed in the G6 group, where the endpoint tumor volume was further reduced to 222.2 ± 78.1 mm^3^, representing an 83.4% inhibition rate. This trend is consistent with the endpoint tumor weight measurements, where the triple‐combination group demonstrates the lowest tumor burden (Figure 5b,d). Despite the intensified therapy, all groups maintain comparable and steadily increasing body weight without obvious weight loss (Figure S12b), suggesting good systemic tolerability of the treatments. The limited antitumor effect observed in the US‐only group compared with PS‐bio + US indicates that US exposure alone is unlikely to be the dominant cause of tumor suppression. Nevertheless, because local tumor temperature was not directly monitored, a minor contribution from acoustic heating cannot be completely excluded.

**FIGURE 5:**
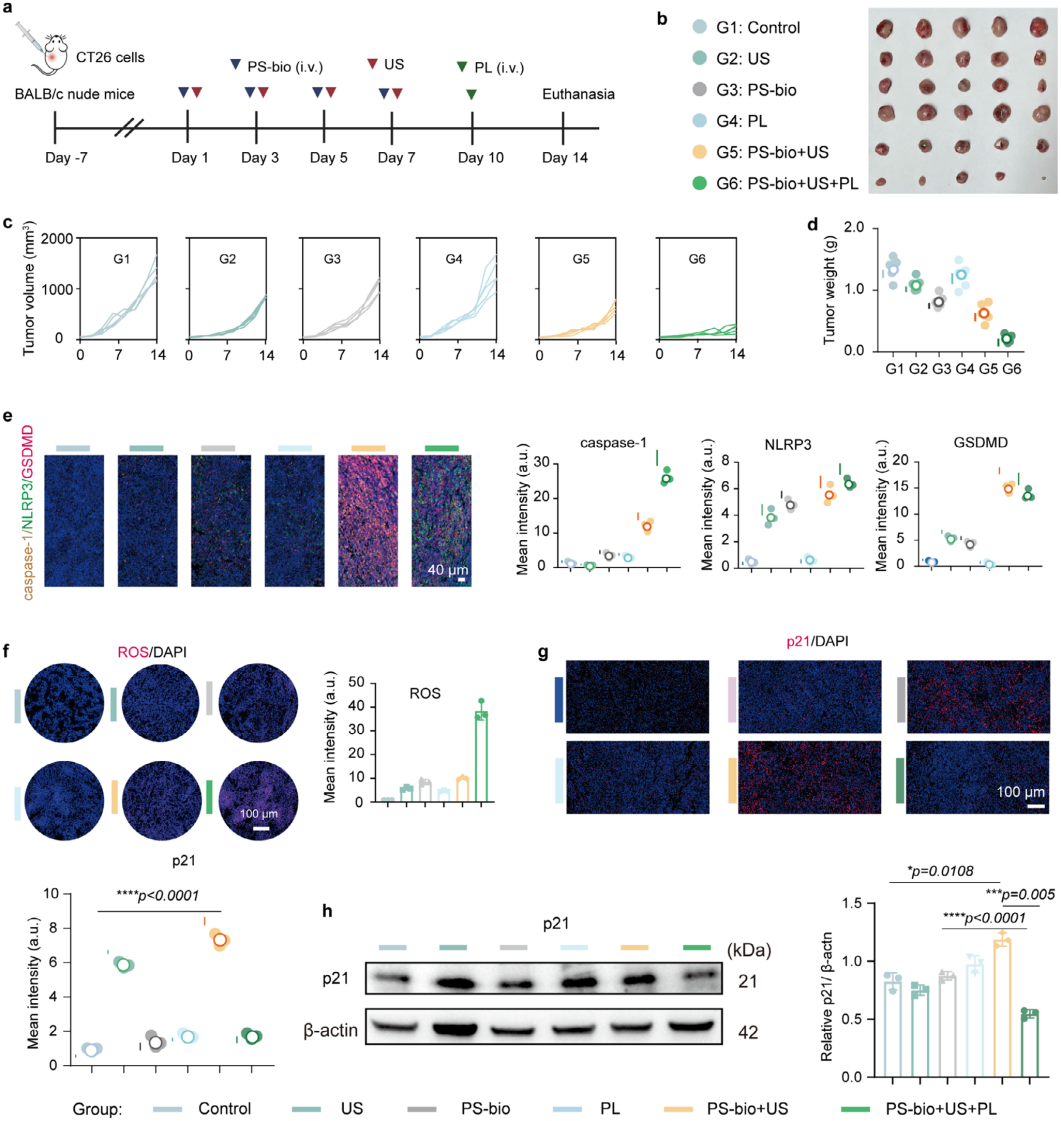
In vivo antitumor effect. (a) Schematic illustration of the treatment timeline in BALB/c nude mice bearing subcutaneous CT26 tumors. Mice were divided into six groups: G1, Control; G2, US; G3, PS‐bio; G4, PL; G5, PS‐bio + US; and G6, PS‐bio + US + PL (n = 5 mice per group). (b) Representative photographs of excised tumors from each group at day 14. (c) Tumor growth curves over time for each treatment group. (d) Tumor weights measured at the experimental endpoint. (e) Representative immunofluorescence images of tumor sections stained for caspase‐1 (gold), NLRP3 (green), and GSDMD (red) and counterstained with DAPI (blue), together with semi‐quantitative analysis of mean fluorescence intensity for each marker. (f) Representative ROS staining images (DCFH‐DA, red) in tumor sections and corresponding quantification of ROS levels. (g) Representative immunofluorescence images of p21 (red) in tumor sections from different groups and semi‐quantitative analysis of p21 intensity. (h) Western blot analysis of p21 expression in tumor lysates and densitometric quantification normalized to β‐actin. Data are presented as mean ± SD. Quantitative data in panels (d–h) were analyzed using one‐way ANOVA followed by Tukey's multiple‐comparison test with *p < 0.05, **p < 0.01, ***p < 0.001, and ****p < 0.0001. The specific groups being compared are indicated by connecting lines in the corresponding graphs.

To further evaluate the impact of the treatments on tumor cell proliferation and histopathology, immunohistochemical staining for Ki67 and hematoxylin & eosin (H&E) staining were conducted. Tumor sections from the control, US‐only, PS‐bio‐only, and PL‐only groups exhibited dense cellular architecture and high Ki67 positivity, indicative of sustained proliferative activity. In contrast, tumors from the PS‐bio + US group shows a noticeable reduction in Ki67 staining intensity (Figure S12c). The weakest Ki67 signal was observed in the PS‐bio + US + PL group, consistent with its most effective suppression of tumor growth. H&E staining results reveal preserved, viable tumor morphology in the control and single‐agent groups. Conversely, tumors treated with PS‐bio + US, and to a greater extent with PS‐bio + US + PL, display extensive areas of cell death, structural disintegration, and necrosis (Figure S12d), providing histopathological evidence that corroborates the enhanced antitumor efficacy of the combination therapies.

We next investigated whether PS‐bio maintained its pyroptosis‐inducing activity in vivo and assessed the influence of subsequent PL treatment on this process. Immunofluorescence analysis of key pathway markers revealed that tumors harvested from mice treated with PS‐bio plus US showed substantially enhanced fluorescence signals for caspase‐1, NLRP3, and GSDMD‐N compared to all other treatment groups (Figure 5e). These findings confirm the robust activation of the inflammasome‐caspase‐1‐GSDMD axis, consistent with the in vitro observations. This pyroptotic signature was largely retained in the PS‐bio + US + PL group, indicating that the subsequent administration of the senolytic agent PL did not attenuate the initial pyroptotic response. To further assess GSDMD activation in vivo, Western blot analysis was performed using frozen tumor tissues from the Control, US, PS‐bio, and PS‐bio + US groups. PS‐bio + US treatment increased the abundance of the cleaved GSDMD‐N fragment compared with the other groups (Figure S12e,f), providing direct biochemical evidence of GSDMD cleavage in tumor tissues and further supporting activation of the pyroptotic pathway in vivo.

Consistent with the catalytic ROS‐generating capacity of PS‐bio, staining for ROS in tumor sections showed only faint fluorescence in the control, US‐only, PS‐bio‐only, and PL‐only groups. In contrast, PS‐bio + US treatment induces intense intratumoral ROS accumulation, an effect that is maintained at similarly high levels in the PS‐bio + US + PL group (Figure 5f). Immunofluorescence and western blot analyses corroborated the cell‐based findings, showing a substantial increase in p21 expression within tumors from the PS‐bio + US group, consistent with the induction of TIS in a residual cell population. Notably, p21 levels are markedly reduced at both the histological and protein levels in the PS‐bio + US + PL group, whereas PL alone shows a minimal effect (Figure 5g,h). Collectively, US‐activated PS‐bio induces robust, ROS‐driven pyroptosis while concurrently generating a p21‐high senescent population. Subsequent administration of PL selectively attenuates this senescent compartment without compromising the pyroptotic response, consistent with its proposed senolytic function and contributing to the enhanced tumor control observed in the CT26 model.

In addition to evaluating antitumor efficacy, we systematically assessed the biosafety profile of the PS‐bio therapeutic regimens. Histopathological examination via H&E staining of major organs (heart, liver, spleen, lungs, and kidneys) collected from all six treatment groups reveals no significant pathological alterations, such as inflammatory infiltration, hemorrhage, or tissue architecture disruption (Figure S13a). Furthermore, a standard in vitro hemolysis assay demonstrates that PS‐bio induced negligible hemolysis across a tested concentration range of 6.25–200 µg mL^−1^. The calculated hemolysis ratios were well below the widely accepted safety threshold of 5% and were comparable to those of the PBS negative control. In contrast, deionized water, used as a positive control, resulted in intensely red supernatants indicative of complete erythrocyte lysis (Figure S13b).

Finally, routine hematology and serum biochemical parameters were monitored to probe potential systemic toxicity. White blood cell counts, red blood cell indices, and platelet‐related parameters in all treatment groups remain within normal ranges and show no significant differences compared with the control group (Figure S14a). Likewise, serum levels of alanine aminotransferase (ALT), aspartate aminotransferase (AST), blood urea nitrogen (BUN), creatinine (CREA), and urea display no statistically significant changes among groups (Figure S14b), suggesting the absence of overt hepatic or renal dysfunction under the current dosing regimen. Although these results support the short‐term tolerability of PS‐bio under the present dosing regimen, the transition‐metal composition of Pd_3_Sn warrants further evaluation of its long‐term biosafety. The current study does not directly determine the long‐term accumulation, degradation, or clearance of Pd and Sn. Future studies incorporating quantitative elemental analysis and extended observation will therefore be needed to further assess the long‐term safety of PS‐bio.

### 2.7 Liver Metastasis Inhibition

Cancer metastasis remains the leading cause of cancer‐related mortality, and patients with distant lesions still have dismal 5‐year survival rates despite advances in systemic therapy [49, 50]. For CRC, the liver is the most frequent site of distant spread and a major determinant of prognosis [51]. In this context, US‐assisted nanotherapeutic strategies have been widely explored to restrain metastatic progression in various models. To further evaluate the translational potential of our strategy in a clinically relevant setting, we established a CRC liver metastasis model using luciferase‐expressing CT26 cells (Figure 6a).

**FIGURE 6:**
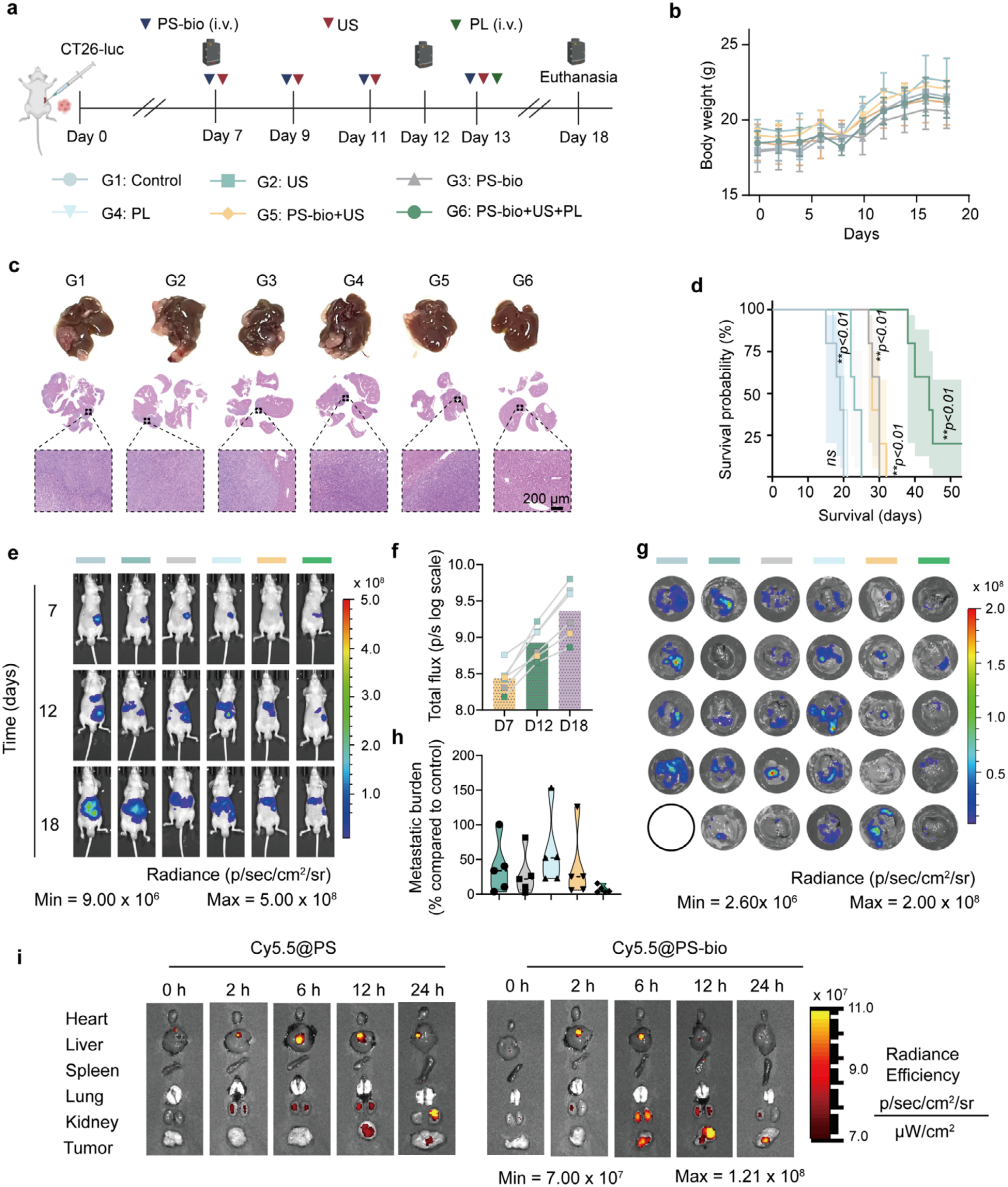
In vivo inhibitory effect of PS‐bio on colorectal cancer liver metastasis. (a) Schematic illustration of the experimental timeline for the liver‐metastasis model and drug/US administration (G1, Control; G2, US; G3, PS‐bio; G4, PL; G5, PS‐bio + US; G6, PS‐bio + US + PL). (b) Body‐weight curves of mice during treatment (n = 5). (c) Representative photographs of livers and corresponding H&E‐stained sections from each group at the experimental endpoint, showing the extent of metastatic nodules. (d) Kaplan‐Meier survival curves of mice receiving different treatments. (e) In vivo bioluminescence imaging of CT26‐luc tumor burden at the indicated time points for each group. (f) Quantitative analysis of in vivo bioluminescence signals, presented as total photon flux at different time points. (g) Ex vivo bioluminescence images of isolated livers at the endpoint, illustrating differences in metastatic load. (h) Quantification of ex vivo metastatic burden, expressed as the percentage of bioluminescence signal relative to the control group. (i) Ex vivo fluorescence imaging of Cy5.5‐labeled PS or PS‐bio at different time points post‐injection, showing biodistribution and tumor accumulation in major organs (heart, liver, spleen, lung, kidney, and tumor). Data in (b), (f), and (h) are presented as mean ± SD (n = 5 mice per group). Body weight (b) and longitudinal bioluminescence data (f) were analyzed using two‐way repeated measures ANOVA followed by Šídák's multiple comparison test. Survival curves in (d) were analyzed using the log‐rank (Mantel–Cox) test. Metastatic burden in (h) was analyzed using one‐way ANOVA followed by Tukey's multiple comparison test. Statistical significance is indicated in the corresponding panels.

Throughout the 18‐day experimental period, all groups maintain comparable body weights without significant loss (Figure 6b), indicating acceptable systemic tolerance. At the experimental endpoint, livers harvested from mice in the control, US‐only, PS‐bio‐only, and PL‐only groups displayed extensive macronodular metastasis, with H&E staining confirming substantial displacement of normal hepatic parenchyma by tumor tissue (Figure 6c). In contrast, animals treated with PS‐bio + US show a significantly reduced metastatic burden, characterized by fewer and smaller tumor foci. Most notably, the PS‐bio + US + PL group demonstrates the most pronounced inhibition of metastasis, with minimal macroscopic tumor infiltration and preservation of normal liver architecture observed histologically, underscoring the superior antimetastatic efficacy of the triple‐combination therapy. The therapeutic impact on animal survival was evaluated by Kaplan‑Meier analysis. Mice in the control and single‑agent groups exhibit rapid mortality, whereas treatment with PS‑bio + US conferred a significant survival advantage. The longest median survival time was observed in the PS‑bio + US + PL group, consistent with the corresponding reduction in hepatic tumor burden (Figure 6d).

To dynamically monitor metastatic progression and treatment response, in vivo bioluminescence imaging (BLI) was performed on days 7, 12, and 18. In the control and US groups, photon flux from the abdominal region increased sharply over time, reflecting aggressive outgrowth of liver metastases (Figure 6e). Treatment with PS‐bio or PL alone resulted in only a modest attenuation of the bioluminescent signal. In contrast, the combination of PS‐bio with US markedly suppressed the increase in total photon flux over time, while the PS‐bio + US + PL group exhibits the lowest signal intensity at all monitored time points. Quantification of the in vivo BLI signal confirms this trend, with the D18 log10‐transformed total photon flux reduced from 9.64 ± 0.33 in the control group to 9.05 ± 0.32 in the PS‐bio + US group and further to 8.86 ± 0.17 in the PS‐bio + US + PL group (Figure 6f). Ex vivo BLI of excised livers at the study endpoint further supports these findings. The control, US, PS‐bio, and PL groups retain strong luminescence, whereas the PS‐bio + US group shows a markedly weakened signal and the PS‐bio + US + PL group displays the lowest ex vivo photon flux (Figure 6g). Corresponding quantitative analysis show that metastatic burden was reduced to 37.9% ± 50.2% in the PS‐bio + US group and further to 6.9 ± 5.4% in the PS‐bio + US + PL group, compared with the control level (Figure 6h).

Although the subcutaneous tumor model provides convenient evaluation of therapeutic efficacy, its superficial location does not fully reproduce the acoustic environment of deep human colorectal lesions. The liver metastasis model provides an additional abdominal treatment setting, but differences in tissue depth, bowel gas, and acoustic attenuation remain important considerations for clinical translation. Image guided focused ultrasound or endoscopic ultrasound, together with adjustment of acoustic parameters according to target depth, may improve energy delivery to deeper lesions. Further validation in clinically relevant deep tissue models will therefore be required before direct translation of the current treatment parameters.

To investigate the in vivo biodistribution and tumor‐associated accumulation of PS‑bio, the tissue localization of Cy5.5‑labeled formulations was tracked using fluorescence imaging. The non‑targeted formulation (Cy5.5@PS) exhibits a relatively diffuse distribution profile with limited specific accumulation in the liver and tumor regions over 24 h (Figure 6i). In contrast, the biotin‑functionalized counterpart (Cy5.5@PS‑bio) rapidly accumulates in the liver and tumor foci within 2–6 h post‑injection, accompanied by moderate fluorescence signals in the spleen and minimal uptake in the heart and kidneys. Hepatic and tumor‐associated fluorescence signals gradually diminish by 24 h, indicating a time‐dependent clearance profile, a favorable characteristic for limiting long‐term metal accumulation in vivo [52]. These fluorescence imaging results support enhanced tumor‐associated accumulation of PS‐bio relative to PS. However, absolute tumor uptake expressed as percentage injected dose (%ID) was not determined because the fluorescence measurements were not calibrated for absolute material quantification. Although PS‐bio shows enhanced tumor‐associated accumulation, fluorescence signals were also detected in the liver. The mildly acidic tumor microenvironment and spatially localized US irradiation may favor ROS generation within tumor regions, while no obvious histopathological abnormalities or alterations in hepatic function were observed under the current treatment regimen. Nevertheless, ROS production was not directly compared between metastatic lesions and adjacent normal liver tissue, and potential off‐target oxidative stress cannot be completely excluded.

Human HT‐29 cells were used for detailed in vitro mechanistic analyses, whereas murine CT26 cells were used to establish reproducible subcutaneous and liver metastasis models. In CT26‐derived tumors, PS‐bio + US increases pyroptosis‐related markers and p21 expression, while subsequent PL treatment reduces the senescence‐associated response, consistent with the overall pattern observed in HT‐29 cells. These findings suggest that the therapeutic response is not limited to a single colorectal cancer model. However, HT‐29 and CT26 cells should not be assumed to exhibit identical molecular responses, and direct comparative validation will be required to determine the extent to which the detailed mechanism is conserved. Because the in vivo experiments were performed in BALB/c nude mice, the present study did not evaluate adaptive antitumor immunity. Accordingly, the inflammatory responses observed here should not be interpreted as evidence of immune‐mediated therapeutic efficacy.

## 3 Conclusion

In summary, we have developed a US responsive sequential therapeutic strategy that effectively orchestrates the pyroptosis‐senescence axis for improved CRC therapy. The rationally designed Pd_3_Sn TQMs exhibit theoretically supported topological semimetal characteristics and facet‐dependent electronic environments that favor peroxide activation, resulting in enhanced sonocatalytic ROS generation and activation of the zDHHC5‐mediated GSDMD pyroptotic pathway. Importantly, PS‐bio plus US treatment also promotes the emergence of therapy induced senescence in surviving cancer cells, while soluble inflammatory factors released after treatment contribute to senescence in neighboring cells. Subsequent administration of the senolytic agent PL reduces the residual senescent cell population and its associated tumor promoting effects, thereby further improving tumor control. Together, these findings demonstrate that sequential pyroptosis induction and senolytic treatment using Pd_3_Sn TQMs represents an effective therapeutic strategy against CRC. This study expands the biomedical application of materials with theoretically supported topological semimetal electronic characteristics and provides a strategy for regulating distinct tumor cell fates through sequential treatment.

## Author Contributions

**Zilu Chen**: methodology, investigation, validation, software, formal analysis, data curation, resources, visualization, Writing – original draft, Writing – review and editing. **Kun Mei**: methodology, investigation, software, data curation, visualization. **Yan Yang**: investigation, methodology, software, visualization. **Mengna Xu**: software, methodology, investigation. **Xinyue Dai**: methodology, investigation, validation, software. **Renjun Gu**: methodology, investigation, validation, visualization. **Fuqiong Zhou**: investigation, methodology, visualization. **Cheng Wang**: methodology, investigation, data curation. **Meiqi Chang**: data curation, conceptualization, investigation, methodology, validation, writing – original draft, funding acquisition, visualization, project administration, supervision. **Yan Huang**: conceptualization, investigation, funding acquisition, writing – original draft, methodology, validation, writing – review and editing, supervision, resources, project administration. **Yu Chen**: conceptualization, investigation, writing – original draft, funding acquisition, methodology, validation, writing – review and editing, formal analysis, project administration, supervision, resources.

## Funding

The work was supported by National Natural Science Foundation of China (No. 52572305), 2024 Nanjing Health Science and technology development special project (No. ZKX24043), 2024 Research project of Provincial Chinese Medicine Society (No. PDJH2024022), Natural Science Foundation of Jiangsu Province (No. BK20240738), General Program of Basic Science (Natural Science) Research of Jiangsu Higher Education Institutions (No. 24KJB360004).

## Conflicts of Interest

The authors declare no conflicts of interest.

## Supporting information




**Supporting File**: advs77968‐sup‐0001‐SuppMat.docx.

## Data Availability

The data that support the findings of this study are available from the corresponding author upon reasonable request.
