# Supplementary material for: Topological Quantum Biomaterials Enable Pyroptosis‐Senescence Coupling for Enhanced Colorectal Cancer Therapy
Source: Adv Sci (Weinh). 2026 Sep 30:e77968. Online ahead of print. doi: 10.1002/advs.77968 (PMC13626165; doi:10.1002/advs.77968)
Supplement: Supplementary file 1 [file ADVS-9999-e77968-s001.docx]

**Supporting Information**

**Topological Quantum Biomaterials Enable Pyroptosis-Senescence Coupling for Enhanced Colorectal Cancer Therapy**

Z*ilu Chen**^1^, Kun Mei^1^, Yan Yang^1^, Mengna Xu**^1^, Xinyue Dai^2^,* *Renjun Gu^4^, Fuqiong Zhou^5^, Cheng Wang^6^, Meiqi Chang^3^*, Yan Huang^1^*, and Yu Chen^2^**

^1^Department of Ultrasound, Nanjing Hospital of Chinese Medicine Affiliated to Nanjing University of Chinese Medicine, Nanjing, 210022, China.

^2^Materdicine Lab, School of Life Sciences, Shanghai University, Shanghai 200444, China.

^3^Science and Technology Innovation Center, Shanghai Municipal Hospital of Traditional Chinese Medicine, Shanghai University of Traditional Chinese Medicine, Shanghai 200071, China.

^4^School of Chinese Medicine, Nanjing University of Chinese Medicine, Nanjing, 210023, China.

^5^Central Laboratory, Nanjing Hospital of Chinese Medicine Affiliated to Nanjing University of Chinese Medicine, Nanjing, 210022, China.

^6^Department of Clinical Laboratory, Nanjing Hospital of Chinese Medicine Affiliated to Nanjing University of Chinese Medicine, Nanjing, 210022, China.

*Corresponding Author: Prof. Meiqi Chang (changmeiqi@vip.sina.com); Prof. Yan Huang (fsyy00243@njucm.edu.cn); Prof. Yu Chen (chenyuedu@shu.edu.cn).

**1.** **Experimental section**

*1.1. Materials*

All chemicals were of analytical purity and used without further purification. Pd(acac)_2_, SnC_2_O_4_, Phloroglucinol, CTAC, Oleylamine, absolute ethanol, CTAB Cyclohexane, NaOH, tetraethyl orthosilicate (TEOS), 3-Triethoxysilylpropylamine (APTES), 3,3′,5,5′-Tetramethylbenzidine dihydrochloride hydrate (TMB) were purchased from Aladdin (Shanghai, China). 5,5-dimethyl-1-pyrroline n-oxide (DMPO), 2,2,6,6-Tetramethyl-4-piperidone hydrochloride (TEMP), Calcein AM/PI Cell Viability/Cytotoxicity Assay Kit, Annexin V-FITC/PI Kit, Cell Counting Kit-8 (CCK-8) were bought from Dojindo (Dojindo, China). JC-1 Mitochondrial Membrane Potential Assay Kit, BCA Protein Assay Reagent Kit, Hoechst 33342, LDH Cytotoxicity Assay Kit (C0016), Cell Cycle and Apoptosis Analysis Kit and ATP Assay Kit was purchased from Beyotime Biotechnology (Shanghai, China). 2,7-dichlorofluorescein diacetate (DCFH-DA) was purchased from MedChemExpress (Shanghai, China). Rhodamine B was purchased from Sigma-Aldrich (California, USA). Mito-Tracker and Lyso-Tracker was obtained from Thermo Fisher Scientific (USA). PBS, McCoy’s 5A medium, DMEM medium, penicillin/streptomycin and fetal bovine serum (FBS) were bought from Gibco Life Technologies Co., Ltd (New York, USA). Human IL-1β ELISA Kit were purchased from Quanzhou Ruixin Biological Technology and Human IL-18 ELISA Kit were purchased from HUABIO.

*1.2. Preparation of Pd_3_Sn nanostructures.*

Pd_3_Sn nanostructures with different morphologies were synthesized *via* a solvothermal method by adjusting the type and amount of structure-directing agents. In a typical procedure, Pd(acac)_2_ (76 mg), SnC_2_O_4_ (17 mg), CTAC (320 mg), and oleylamine (50 mL) were added into a reaction flask and sonicated for 60 min to form a homogeneous solution. The mixture was then heated from 30 °C to 170 °C in an oil bath and maintained for 2 h. After naturally cooling to room temperature, the products were collected by centrifugation and washed three times with ethanol. When 500 mg of phloroglucinol was added as the structure-directing agent, the obtained sample was denoted as Pd_3_Sn-Network (Pd_3_Sn-N). When phloroglucinol was replaced by 300 mg of glucose under the same conditions, the sample was labeled as Pd_3_Sn-Strip (Pd_3_Sn-S). For the synthesis of Pd_3_Sn-Dots (Pd_3_Sn-D), the amounts of glucose and CTAC were increased to 600-1200 mg and 960-1920 mg, respectively, while other parameters were kept unchanged. All obtained samples were washed with ethanol three times and dried under vacuum for further use.

*1.3. Synthesis of Pd_3_Sn-biotin (PS-bio).*

CTAB (100 mg) was dissolved in 20 mL of water, and 50 mg of Pd_3_Sn material pre-dispersed in cyclohexane was added to this solution. The mixture was sonicated for 20 min and then vigorously stirred at room temperature for 2 h to allow complete evaporation of cyclohexane. Subsequently, 0.1 M NaOH was added to adjust the pH of the suspension to 10. A mixture of tetraethyl orthosilicate (TEOS, 100 μL) and ethanol (1 mL) was subsequently added dropwise to the aforementioned mixture in 50 μL aliquots at 30-minute intervals. Upon completion of the addition, the reaction mixture was stirred at room temperature for an additional 12 h. Next, 50 μL of APTES was added and the mixture was further stirred for 30 min. Finally, the product was collected by centrifugation, and the precipitate was washed three times with water/ethanol to remove residual reagents. After centrifugation and washing, the amino-functionalized product was redispersed and reacted with biotin-PEG-NHS at room temperature overnight. Finally, the product was collected by centrifugation and washed three times with water/ethanol to remove unreacted reagents, yielding biotin-functionalized Pd_3_Sn nanostructures, denoted as PS-bio.

*1.4. Characterization.*

Transmission electron microscope (TEM) and HRTEM observation JEOL JEM-2100 microscope and Gatan CCD camera were used to record images. The crystalline structure of materials was recorded by XRD (Rigaku Ultima IV, Japan) in the 2θ range of 10°-80°. Solid UV-vis diffuse reflectance spectra were recorded using a UV-vis spectrophotometer with BaSO_4_ as the reference. The optical band gaps were estimated from Tauc plots after Kubelka-Munk transformation of the reflectance spectra. Element mapping is captured by JEM-ARM 300F Grand ARM (JEOL Company Ltd, Japan). XPS measurements were performed on Thermo Scientific K-Alpha (USA) provided by eceshi (www.eceshi.com). The absorbance was measured using a Spectra M2 microplate reader (Molecular Devices, CA, USA). The fluorescent images were captured by confocal laser scanning microscopy (CLSM, CarlZeiss LSM710, ZEISS, Germany). The DCFH-DA and cell cycle analysis was evaluated by flow cytometry (FACS Canto II, Molecular Devices, USA). The change of dissolved oxygen level was monitored by a dissolved oxygen meter (INESA Scientific Instrument, Shanghai). Zeta potential measurements were performed through Mastersizer 3000 (Malvern, Britain). Fourier transform infrared (FT-IR) spectra were conducted on a FT-IR spectrophotometer (Thermal Scientific Nicolet iS20). ESR spectra were acquired by a Bruker EMX1598 spectrometer. Crystal processing software-Diamond, is used to exhibit crystal information and structure. ECL imaging was performed in a Tanon 5200 chemiluminescence imaging system.

*1.5. Colloidal stability of PS-bio.*

PS-bio was dispersed in PBS or culture medium containing 10% fetal bovine serum at a concentration of 100 μg mL⁻¹ and incubated at 37 °C. The hydrodynamic diameter and polydispersity index (PDI) were measured by dynamic light scattering at 0, 6, 12, 24, and 48 h. Measurements were performed using three independently prepared samples.

*1.6. Linear Sweep Voltammetry (LSV) Measurement.*

The ROS-related electrochemical activity of Pd_3_Sn nanostructures (Dots, Strip, and Network) were evaluated by LSV using a CHI660E electrochemical workstation (Chenhua, Shanghai, China). All measurements were performed in 0.1 M acetate buffer at pH 5.5. The working electrode was prepared by drop-casting the Pd_3_Sn sample onto a clean glassy carbon electrode and drying under ambient conditions. A platinum wire and an Ag/AgCl electrode were used as the counter and reference electrodes, respectively. LSV scans were recorded from -0.6 V to 0.2 V at a scan rate of 50 mV s^-1^. For H₂O₂-assisted measurements, 5 mM H₂O₂ was added to the electrolyte immediately before data acquisition. All experiments were conducted at room temperature, and each measurement was repeated at least three times to ensure reproducibility.

*1.7. First-principles calculations and topological surface-state analysis.*

Density functional theory (DFT) calculations were carried out using the Vienna Ab initio Simulation Package (VASP)^[1]^.The exchange-correlation energy was treated within the generalized gradient approximation (GGA) using the Perdew-Burke-Ernzerhof (PBE) functional^[2]^. The projector augmented-wave (PAW) method was employed to describe the ion–electron interaction^[3-4]^. A plane-wave kinetic energy cutoff of 350 eV was used. Brillouin-zone integrations were performed with a Monkhorst-Pack k-point mesh of 12 × 12 × 12^[5]^. Electronic self-consistency was reached when the total energy change was below 10⁻⁶ eV. Geometry optimizations were performed until the residual Hellmann-Feynman force on each atom was less than 0.01 eV Å⁻¹. Spin-orbit coupling (SOC) was included to account for relativistic effects^[6]^. To analyze surface-state features, maximally localized Wannier functions were constructed using Wannier90^[7]^, and the surface spectral functions were obtained using WannierTools based on the Wannier tight-binding Hamiltonian^[8]^.

For the additional topological analysis, Wannier Hamiltonians were constructed using the Sn s and p orbitals and the Pd s, p, and d orbitals. The resulting Hamiltonians contained 31 orbitals without SOC and 62 spinor orbitals with SOC. The Wannier interpolation was checked against the corresponding DFT eigenvalues in the energy range around the Fermi level. A refined momentum scan was used to locate the nodal crossing between Wannier bands 13 and 14 along the M–Γ direction, and the Berry phase was calculated along a closed loop enclosing this crossing.

The Zak phase along the [111] direction was calculated by integrating the non-Abelian Berry connection on a 21 × 21 projected momentum mesh using 201 integration points along each path. Projected momentum points with a direct gap ≤ 1 meV were excluded from the Zak phase analysis. For the high precision SOC calculation, a plane-wave cutoff of 450 eV, a 10 × 10 × 10 Γ-centered k-point mesh, and an electronic convergence criterion of 10^-8^ eV were used to examine the robustness of the semimetallic electronic structure. Semi-infinite surface Green-function calculations were further performed for the Pd_3_Sn (111) and (100) terminations. The (100) termination corresponds to the same crystallographic surface orientation previously indexed as the (200) lattice plane in the experimental characterization.

*1.8 DPBF degradation assay for US-enhanced ROS generation*

The US-enhanced ROS generation ability of Pd₃Sn nanostructures was evaluated using 1,3-diphenylisobenzofuran (DPBF) as a ROS probe. Briefly, DPBF was mixed with Pd_3_Sn-Dots, Pd_3_Sn-Strip, or Pd_3_Sn-Network dispersions, followed by treatment in the dark or under US irradiation. The decrease in DPBF absorbance at 410 nm was monitored at predetermined time intervals using a UV-vis spectrophotometer. The degradation kinetics were fitted according to a pseudo-first-order model, and the apparent rate constants under dark and US conditions were defined as *k_dark_* and *k_US._*.

*1.9. Peroxidase (POD)-like activity.*

The POD-like activity assay of the prepared samples was performed using TMB as the POD substrate with the assistance of H_2_O_2_. In a typical test, TMB (0.8 mM), H_2_O_2_ (1 mM), and PS-bio (100 μg mL^−1^) were mixed in deionized water. As the reaction proceeded, the blue color was developed and the absorbance changes of the reaction mixture at 652 nm were measured through a microplate reader.

*1.10. Catalase (CAT)-like activity.*

The CAT-like activity was evaluated based on the O_2_ generation in the presence of different concentrations of H_2_O_2_ (0, 12.5, 25, 50, and 100 mM). Typically, 100 μg ml^-1^ samples was added into deionized water with H_2_O_2_, the concentration of O_2_ was monitored for 400 seconds by using dissolved oxygen meter (INESA Scientific Instrument, Shanghai).

*1.11. Oxidase (OXD)-mimic activity.*

TMB as the substrate was used to measure the OXD-mimic activity of samples. Typically, TMB (300 μg mL^−1^) and PS-bio (100 μg mL^−1^) were dispersed in PBS (3 mL), and the absorbance was measured after a certain reaction time by using a UV spectrophotometer.

*1.12. Electron spin resonance (ESR) measurement.*

The experiment was divided into four groups: (1) control, (2) US (1 MHz; 1.2 W cm^−2^; 50% duty cycle; 1 min), (3) PS-bio, (4) PS-bio + US. •OH, •O_2_^-^ and ^1^O_2_ can be captured by 5,5-dimethylpyrroline N-oxide **(**DMPO) and 2,2,6,6-tetramethylpiperidine (TEMP) and detected by an electron paramagnetic resonance spectrometer.

*1.13. DFT calculations for H_2_O_2_ activation on representative Pd_3_Sn facets.*

DFT as implemented in the VASP was employed to optimize geometry structures^[1]^. The exchange-correlation interactions were described by the GGA in the form of the PBE^[2]^. We have chosen the PAW potentials^[3]^ to describe the ionic cores and take valence electrons into account using a plane wave basis set with a kinetic energy cutoff of 450 eV. The electronic energy was considered self-consistent when the energy change was smaller than 10^-5^ eV. A geometry optimization was considered convergent when the force change was smaller than 0.04 eV/Å. The vacuum spacing in a direction perpendicular to the plane of the structure is 15 Å. The van der Waals interactions were considered by the method of the Grimme (DFT+D3)^[9]^. The Brillouin-zone integration was sampled with a Monkhorst-Pack mesh of 2× 2 × 1 in the structural relaxation calculations.

The Gibbs free energy change (Δ*G*) of a gas phase molecule or an adsorbate on the surface was calculated based on Nørskov’s computational hydrogen electrode (CHE) method^[10]^ by the equation:

ΔG = ΔE + ΔE_ZPE_ - TΔS

where ΔE is the total energy change directly obtained from DFT calculations, ΔE_ZPE_ is the zero-point energy change, ΔS is the entropy correction change, T is the temperature which is normally estimated at 298.15 K.

*1.14. Cell culture.*

Human colon carcinoma cell (HT-29) was purchased from Haixing Biosciences, and normal human colonic epithelial cells (NCM460) were purchased from Procell Life Science & Technology Co., Ltd. Mouse colon carcinoma cells (CT-26 and CT26-luc) were purchased from the Shanghai QuiCell Biotechnology. HT-29, CT-26, and CT26-luc cells were cultured in high glucose DMEM or McCoy’s 5A medium supplemented with 10% fetal bovine serum and 1% penicillin/streptomycin. NCM460 cells were cultured in NCM460 Cell Complete Medium (Procell, CM-0749). All cells were cultured at 37 °C with 5% CO_2_.

*1.15. Cellular uptake.*

We evaluated the cellular internalization process of PS-bio by confocal laser scanning microscope (CLSM) observation. 3D spheroids were formed using 3D Cell Culture Coating Kit (Beyotime, C0366S). Rhodamine B-labeled PS-bio was incubated with the cells for different periods (1, 2 and 4 h). Then the cells were washed by PBS and stained with Hoechst 33342. The treated cells were then visualized by CLSM. In lysosome phagocytosis assay, HT-29 cells were seeded into confocal dishes and cultured overnight. Rhodamine B-labeled PS-bio was then incubated with the cells for different time periods (0, 2, 4, 6, 12 and 24 h). Subsequently, the cells were stained with Hoechst 33342 and Lyso-Tracker Green (Thermo). The treated cells were then visualized by CLSM. Colocalization between LysoTracker and Rhodamine B-labeled nanoparticles was quantitatively analyzed using Fiji/ImageJ with the Coloc 2 plugin. Individual cells were delineated as regions of interest (ROIs), and the same ROI was applied to both fluorescence channels. Pearson’s correlation coefficient (PCC, Pearson’s r without thresholding) was calculated to assess the pixel-by-pixel correlation between the LysoTracker and nanoparticle fluorescence signals. Data are presented as mean ± SD. Differences among multiple time points were analyzed by one-way ANOVA followed by Tukey’s multiple-comparisons test. A P value < 0.05 was considered statistically significant.

*1.16. In vitro cytotoxicity of PS-bio.*

Cytotoxicity of PS-bio was evaluated with a cell counting kit-8 (CCK-8) assay. Briefly, HT-29 cells were seeded into 96-well plates at a density of 5 × 10^4^ cell mL^-1^ and incubated overnight at 37 °C. The cell culture medium was refreshed with fresh medium containing different concentrations of PS-bio (0, 50, 100, 200, 400 μg mL^-1^). US irradiation (1.0 MHz, 1.0 W cm^-2^, 50% duty cycle, 2 min) was applied in the designated group after co-incubation. Then, cytotoxicity was measured by the CCK-8 method.

*1.17. Cell live/death detection.*

HT-29 cells were seeded in 6-well plates and cultured overnight. Then the experiment was divided into the following four groups: 1. Control; 2. US (1.0 W cm^-2^, 50 % duty cycle, 2 min); 3. PS-bio (50 µg mL^-1^); 4. PS-bio + US. US irradiation was proceeded in the designated group after 6 h PS-bio incubation. Then, calcein AM/PI cell viability/cytotoxicity assay kit was used to treat the cells. Finally, HT-29 cells were washed twice with PBS, and then imaged using a fluorescent microscope.

*1.18. Detection of intracellular ROS.*

HT-29 cells were seeded in 6-well plates and cultured overnight. Then the experiment was divided into the following four groups: 1. Control; 2. US (1.0 W cm^-2^, 50 % duty cycle, 2 min); 3. PS-bio (50 µg mL^-1^); 4. PS-bio + US. Subsequently, the cells were washed with PBS and incubated with DCFH-DA (10 μM) at 37 °C for 25 min, followed by Hoechst 33342 staining for another 10 min. Then, the fluorescence intensity was assessed *via* CLSM with Ex/Em of 488/525 nm. At last, intracellular fluorescence was observed through fluorescent microscope.

*1.19. Detection of mitochondrial ROS.*

HT-29 cells were seeded in 6-well plates and cultured overnight. Then the experiment was divided into the following four groups: 1. Control; 2. US (1.0 W cm^-2^, 50 % duty cycle, 2 min); 3. PS-bio (50 µg mL^-1^); 4. PS-bio + US. Subsequently, the cells were washed with PBS and incubated with Mito-SOX Red staining (5 μM) at 37 °C for 30 min, followed by Mito-tracker green and Hoechst 33342 staining for another 10 min. Then the cells were washed with PBS and observed by CLSM.

*1.20. Detection of mitochondrial membrane potential.*

HT-29 cells were seeded in a confocal glass bottom-dish (cell density = 10^5^ cells per disk) and cultured overnight. Then the experiment was divided into the following four groups: 1. Control; 2. US (1.0 W cm^-2^, 50 % duty cycle, 2 min); 3. PS-bio (50 µg mL^-1^); 4. PS-bio + US. The US-related groups were exposed to the US irradiation and co-incubation at 37 °C for another 6 h. Then the cells were stained with JC-1 mitochondrial membrane potential assay kit according to the standard procedure for 20 min at 37 °C. Then the cells were washed with PBS and observed by CLSM (the Ex/Em of JC-1 monomer and polymer were 490/530 nm and 525/590 nm, respectively).

*1.21. RNA sequencing.*

Then the experiment was divided into the following two groups: 1. Control; 2. PS-bio (20 µg mL^-1^) + US (1.0 W cm^-2^, 50 % duty cycle, 2 min). The mRNA sequencing was performed in Shanghai Majorbio Bio-Pharm Technology Co., Ltd. The data were analyzed online by using the free platform of Shanghai Majorbio Bio-Pharm Technology Co., Ltd (<https://cloud.majorbio.com/>).

*1.22. Analysis of pyroptosis.*

HT-29 cells were seeded in 6-well plates and cultured for 24 h up to about 80 % confluence. The experiment was divided into the following four groups: 1. Control; 2. US (1.0 W cm-2, 50 % duty cycle, 2 min); 3. PS-bio (50 µg mL-1); 4. PS-bio + US. The cell pyroptosis can be observed in the bright field and imaged using a field-emission SEM (Hitachi SU8100, Japan) operated at an accelerating voltage of 3.0 kV. Pyroptotic morphology, including membrane blebbing and balloon-like protrusions, was compared among the different treatment groups.

*1.23. Cell viability assay of piperlongumine (PL)-treated senescent cells.*

HT-29 cells were treated with 200 μM H₂O₂ for 2 h and then cultured in fresh complete medium for 48 h to induce a senescence like phenotype. Normal and H₂O₂-induced senescent HT-29 cells were subsequently treated with PL at 0, 5, 10, 15, 20, and 25 μM for 24 h. Cell viability was measured using the CCK-8 assay, and absorbance was recorded at 450 nm. Relative viability was normalized to the corresponding 0 μM PL group.

*1.24. Cellular senescence detection and senescent cell clearance.*

HT-29 cells were seeded in 6-well plates and cultured overnight. Then the experiment was divided into the following five groups: 1. Control; 2. US (1.0 W cm^-2^, 50 % duty cycle, 2 min); 3. PS-bio (50 µg mL^-1^); 4. PS-bio + US; 5. PS-bio + US+PL. Then, the cells were fixed with SA-β-Gal staining stationary solution (Solarbio, G1580) at room temperature for 15 min. After washing three times with PBS, the pre-prepared SA-β-Gal dyeing working liquid was added and incubated overnight at 37 °C. Then the cells were washed with PBS and observed by microscope. For cell-cycle analysis, the treated HT-29 cells were harvested by trypsinization, collected by centrifugation, and washed twice with cold PBS. The cell pellets were fixed in 70% ice-cold ethanol at 4 °C overnight. After fixation, cells were washed with PBS and resuspended in PI/RNase staining solution (50 µg mL^-1^ propidium iodide and 100 µg mL^-1^ RNase A in PBS). Following incubation for 30 min at 37 °C in the dark, DNA content was analyzed by flow cytometry, and the percentages of cells in G0/G1, S and G2/M phases were calculated using FlowJo software.

*1.25. Quantiﬁcation of in vitro cytokines.*

To collect the cell culture supernatant and centrifuge at 800 rpm for 5 minutes at 4 °C to remove cell culture debris. The levels of cytokines in the supernatant were analyzed using a Proteome Profiler Human XL Cytokine Array (R&D systems, ARY022B).

*1.26. Cellular immunofluorescence staining.*

HT-29 cells were seeded in a confocal glass bottom-dish (cell density = 10^5^ cells per disk) and cultured overnight. Then the experiment was divided into the following four groups: 1. Control; 2. US (1.0 W cm^-2^, 50 % duty cycle, 2 min); 3. PS-bio (50 µg mL^-1^); 4. PS-bio + US. Then, cellular immunofluorescence staining experiments of γ-H2AX were carried out. The treated cells were then visualized by CLSM.

*1.27. Western blot analysis.*

HT-29 cells were seeded in 6-well plates and cultured for 24 h up to about 80 % confluence. The experiment was divided into the following four groups: 1. Control; 2. US (1.0 W cm^-2^, 50 % duty cycle, 2 min); 3. PS-bio (50 µg mL^-1^); 4. PS-bio + US. Cells were washed twice with PBS and collected using a cell scraper. Then, the cells were digested with RIPA lysate on ice for 30 min, and the protein solution was obtained by centrifugation. Subsequently, the protein concentration was determined by BCA protein assay kit. Finally, standard western blot was performed. Anti-Caspase 1 and cleaved-Caspase 1 antibody (abmart), anti-NLRP3 antibody (abmart), anti-GSDMD antibody (HUABIO), Anti-p21 antibody (CST), Anti-zDHHC5 antibody (HUABIO) were applied. Finally, the protein was detected by Bio-Rad ChemiDoc MP and the protein expressions were quantified by software Image J.

*1.28. Lactate dehydrogenase (LDH) assays.*

HT-29 cells were seeded in 6-well plates and cultured for 24 h up to about 80 % confluence. The experiment was divided into the following four groups: 1. Control; 2. US (1.0 W cm^-2^, 50 % duty cycle, 2 min); 3. PS-bio (50 µg mL^-1^); 4. PS-bio + US. Finally, the release of LDH was detected according to the instructions of LDH cytotoxicity test kit.

*1.29. Detection of intracellular adenosine triphosphate (ATP).*

HT-29 cells were seeded in 6-well plates and cultured for 24 h up to about 80 % confluence. Then the experiment was divided into the following four groups: 1. Control; 2. US (1.0 W cm^-2^, 50 % duty cycle, 2 min); 3. PS-bio (50 µg mL^-1^); 4. PS-bio + US. The supernatant was collected by lysing cells and then added to the ATP assay kit. ATP content was calculated by the luminescence intensity measured by a BioTek Synergy4 multifunctional plate reader.

*1.30. Interleukin-1β (IL-1β) and IL-18 secretion.*

HT-29 cells were seeded in 6-well plates and cultured for 24 h up to about 80 % confluence. The experiment was divided into the following four groups: 1. Control; 2. US (1.0 W cm^-2^, 50 % duty cycle, 2 min); 3. PS-bio (50 µg mL^-1^); 4. PS-bio + US. Then the release of IL-1β and IL-18 was detected by the Human IL-1β ELISA kit and Human IL-18 ELISA kit.

*1.31. Conditioned medium transfer assay.*

HT-29 donor cells were assigned to the Control and PS-bio + US groups. After treatment, the original medium was removed, and the cells were thoroughly washed before fresh complete medium was added. Conditioned medium was subsequently collected and applied to untreated recipient HT-29 cells. Senescence in recipient cells was evaluated by SA-β-gal staining.

*1.32. Acyl biotin exchange (ABE).*

ABE was performed according to a previously described procedure with modifications^[11]^. Cells were treated and collected as specified and centrifuged (12,000 g, 20 min). Cell pellets were resuspended in 0.2 ml buffer A (50 mM Tris-HCl pH 7.4, 1 mM EDTA, 150 mM NaCl, 1% NP-40) with 50 ml N-ethylmaleimide (NEM, Targetmol, T3088-10mg) and protease inhibitor cocktail. The samples were incubated for 2 h with gentle rotation at 4 °C and centrifuged at 20,000 g for 20 min. The supernatant was collected and the BCA Protein Assay Kit was used to determine the protein concentration to equalize the amount of protein in each sample.

The sample was then precipitated with a methanol-chloroform-water mixture. In brief, 800 μL methanol, 300 μL chloroform and 600 μL double-distilled H_2_O were added to each sample sequentially with thorough mixing by vortexing at each step. The mixtures were centrifuged for 5 min at 16,000 g in a microfuge and the top aqueous layer was discarded. Then, 800 μL methanol was added to resuspend each pellet. The mixtures were thoroughly vortexed and centrifuged for 5 min at 16,000 g, and the supernatants were removed as much as possible. The pellets were air-dried for 20-30 min. The above methanol-chloroform precipitation was repeated two additional times.

The dried pellets were resuspended in 0.2 ml buffer B (50 mM Tris-HCI pH 7.4, 5 mM EDTA 150 mM NaCl. 1% NP-40,1% SDS) with or without 0.7 M HAM (Aladdin, H164487-25 mL) to totally dissolve the pellets by pipetting or even water sonication and incubated with gentle rotation at room temperature for 1 h. The samples were precipitated using the same above methanol-chloroform protocol and dissolved in buffer B with 4mM *N*-6-(biotinamido) hexyl]-3-(2'-pyridyldithio) propionamide (Biotin HPDP, Shanghai YuanYe Bio-Technology Co., Ltd, S25579-10 mg) and incubated with gentle rotation at room temperature. After incubation for 1 h, the samples were precipitated by the same above methanol-chloroform protocol three times. After drying, the pellets were resuspended in 0.1 mL buffer B, and a certain percentage of each sample was taken out as a loading control. The SDS concentration in each remaining sample was diluted tenfold to 0.1% by buffer C (50 mM Tris-HCI pH 7.4, 1 mM EDTA, 150 mM NaCl) before adding 30 μL prewashed Streptavidin agarose (Beyotime Biotechnology, P2159-1 mL). The mixture was incubated overnight at 4 °C with rotation. The agarose beads were washed three times using buffer C with 0.1% NP-40 and 0.1% SDS, mixed with 2x SDS-loading buffer containing β-mercaptoethanol and incubated at 98 °C for 5 min before running an SDS-PAGE. The gels were then transferred and immunoblotted with GSDMD antibody.

*1.33. siRNA mediated knockdown of zDHHC5.*

HT-29 cells were seeded in six well plates and cultured until approximately 50–60% confluence. Three independent small interfering RNAs targeting human zDHHC5 were transfected using jetPRIME^®^ transfection reagent (Polyplus-transfection, France) according to the manufacturer’s instructions. A nonspecific siRNA was used as the negative control. The three zDHHC5 siRNA sequences were as follows: siZDHHC5-1, sense 5′-GGACUAAGCCUGUAUGUGU (dT)(dT)-3′ and antisense 5′-ACACAUACAGGCUUAGUCC (dT)(dT)-3′; siZDHHC5-2, sense 5′-GUCACAAUGGCAGUAAUGU (dT)(dT)-3′ and antisense 5′-ACAUUACUGCCAUUGUGAC (dT)(dT)-3′; and siZDHHC5-3, sense 5′-GAAGACAAUUGUAAUCAGA (dT)(dT)-3′ and antisense 5′-UCUGAUUACAAUUGUCUUC (dT)(dT)-3′. After transfection, knockdown efficiency was evaluated by RT-qPCR and western blotting. The siRNA showing the strongest reduction in ZDHHC5 expression was selected for subsequent ABE analysis.

*1.34. RNA extraction and quantitative real time PCR.*

Total RNA was extracted from HT-29 cells using the EZ-press RNA Purification Kit (EZBioscience, USA, B0004D) according to the manufacturer’s instructions. RNA concentration and purity were determined before reverse transcription. Genomic DNA was removed and cDNA was synthesized using HiScript III RT SuperMix for qPCR (+gDNA wiper) (Vazyme Biotech, Nanjing, China, R323-01). Briefly, RNA was treated with gDNA wiper at 42 °C for 2 min, followed by reverse transcription at 37 °C for 15 min and enzyme inactivation at 85 °C for 5 s.

Quantitative PCR was performed using ChamQ Universal SYBR qPCR Master Mix (Vazyme Biotech, Q711-02) in a 20 μL reaction system. The amplification program consisted of initial denaturation at 95 °C for 30 s, followed by 40 cycles of 95 °C for 10 s and 60 °C for 30 s, followed by melting curve analysis. The primer sequences for ZDHHC5 were 5′-AGTCTAAGGGAAGCCTGGAGATA-3′ (forward) and 5′-CTGACGTACTGCTGCTACTGTAA-3′ (reverse). The primer sequences for GAPDH were 5′-GGAGCGAGATCCCTCCAAAAT-3′ (forward) and 5′-GGCTGTTGTCATACTTCTCATGG-3′ (reverse). GAPDH was used as the internal reference gene, and relative ZDHHC5 mRNA expression was calculated using the 2^−ΔΔCt method.

*1.35. Molecular docking.*

Molecular docking was performed using AutoDock Vina 1.1.2 to evaluate the binding of Piperlongumine to CDKN1A (UniProt: P38936) and CDKN2A (UniProt: P42771). The 3D structure of Piperlongumine (PL) was obtained from PubChem and energy-minimized in ChemDraw 20.0. Protein models of CDKN1A_P38936_human and CDKN2A_P42771_human were downloaded from the AlphaFold Protein Structure Database. Protein preparation, including removal of water molecules and addition of hydrogen atoms, was carried out in PyMOL 2.4. Ligand and protein files were converted to PDBQT format using AutoDock Tools 1.5.6. The docking grid was set to cover the entire protein, and other parameters were kept at default settings. For each docking, 10 binding poses were generated. The conformation with the lowest binding energy was selected as the optimal binding mode. Docking was repeated three times, and the mean binding energy was calculated. The docking results were visualized using PyMOL 2.4 and Discovery Studio 2019.

*1.36. Hemolysis Assay.*

This method is a general approach for evaluating the blood compatibility of nanoparticles. Mouse whole blood was mixed with PBS and centrifuged; after repeating this process several times, the blood cells were resuspended in PBS. A 50 μL aliquot of the cell suspension was mixed with an equal volume of PS-bio at different concentrations. PBS group served as the negative control, and ddH_2_O group as the positive control. After standing for 4 h, the mixture was centrifuged, and the supernatant was collected to measure the absorbance at 542 nm.

*1.37. Human Specimens.*

This study, which retrospectively collected tissue samples from patients, received ethical approval from the Ethics Committee of Nanjing Hospital of Chinese Medicine and registered under ethical review acceptance No. 2026KY013-01. All procedures involving human materials were conducted in accordance with the Declaration of Helsinki and relevant institutional guidelines. Written informed consent was obtained from all participants or their legal guardians prior to specimen inclusion.

*1.38. Animals.*

All animal experiments in this study were in compliance with relevant ethical norms and were approved by the Laboratory Animal Ethics Committee of Nanjing Hospital of Traditional Chinese Medicine Affiliated to Nanjing University of Chinese Medicine (IRM-DWLL-2023028). 5-week-old BALB/c male nude mice were purchased from Gempharmatech C. All animals were raised together under standard conditions (23-26 °C, 40-60% humidity, 12-hour light-dark cycle, 5 per cage), and were allowed to eat and drink freely. When the tumor burden exceeds 10% of the animal's own weight, its weight drops by 20% of its normal weight, or ulcers appear at the tumor site, the experimental animal should be euthanized. None of the experimental animals in this study exceeded the maximum tumor burden.

*1.39. In vivo antitumor effect.*

BALB/c male nude mice were subcutaneously inoculated with 5×10^6^ CT26 cells in 100 μL sterile PBS. When tumors reached 50 mm^3^, mice were randomly assigned to six groups (each n = 5): G1, Control; G2, US; G3, PS-bio; G4, PL; G5, PS-bio+US; and G6, PS-bio+US+PL. PS-bio was injected via the tail vein on days 1, 3, 5 and 7 at 15 mg kg^−1^. Ultrasound irradiation was applied to the tumor 0.5 h after each PS-bio injection (1.2 W cm^−2^, 50% duty cycle, 1.5 min). To minimize transducer-related heat accumulation during repeated treatments, two identical ultrasound devices were used alternately, allowing cooling between successive irradiations. Mice in the US group received the same ultrasound treatment without PS-bio. PL was administered intravenously on day 10 at 18 mg kg^−1^ in the PL and PS-bio+US+PL groups. Then the mice were sacrificed, the tumors of the mice were collected for photography, and they were preserved under appropriate conditions for subsequent testing. Tumor tissue sections were subjected to H&E staining, immunohistochemical staining and immunofluorescence staining.

*1.40. In vivo anti-metastasis efficacy.*

Five-week-old male BALB/c nude male mice were purchased from Gempharmatech C., Mice were anesthetized, and a small left subcostal incision was made to expose the spleen. CT26-luc cell (3×10^5^) suspended in 100 μL sterile PBS were slowly injected into the splenic parenchyma using a 29 G insulin syringe. Gentle pressure was applied for several minutes to prevent backflow, after which the spleen was returned to the abdominal cavity and the incision was sutured. Mice were monitored until full recovery.

Seven days after tumor cell inoculation, mice with comparable bioluminescence signals were randomly divided into six groups (G1-G6, n=5 per group): G1, Control; G2, US; G3, PS-bio; G4, PL; G5, PS-bio+US; and G6, PS-bio+US+PL. PS-bio was administered *via* tail vein injection on days 7, 9, 11 and 13 at a dose of 15 mg kg^−1^. US irradiation was applied transabdominally to the anatomical region corresponding to the liver 0.5 h after each PS-bio injection using a therapeutic ultrasound device (1.2 W cm⁻², 50% duty cycle, 1.5 min). Ultrasound coupling gel was applied between the probe and the abdominal skin to ensure acoustic coupling. PL was injected intravenously on post-inoculation day 13 at a dose of 18 mg kg^−1^ in the PL and PS-bio+US+PL groups. Body weight was recorded every 2 days throughout the treatment period. For survival analysis, mice were observed until they reached humane endpoint criteria, and survival curves were generated using the Kaplan-Meier method. The liver metastasis of mice was observed on post-inoculation day 7, 12 and 18 respectively by using the in vivo IVIS spectral imaging system (PerkinElmer). Then the mice were sacrificed, and the liver tissue was taken out for digital photography and H&E staining.

To evaluate the *in vivo* biodistribution and tumor-targeting ability of the nanoformulation, PS and PS-bio were labeled with Cy5.5 (Cy5.5@PS and Cy5.5@PS-bio). At 0, 2, 6, 12, and 24 h post-injection, mice were anesthetized and imaged using the IVIS system with an excitation/emission filter set appropriate for Cy5.5. After the final imaging time point, mice were sacrificed, and major organs (heart, liver, spleen, lung, kidney) and tumors were harvested and subjected to *ex vivo* fluorescence imaging. The fluorescence intensity in each organ was quantified using the instrument software to compare the accumulation and clearance behavior of Cy5.5@PS and Cy5.5@PS-bio.

*1.41. Safety Evaluation.*

After mice sacrificed, blood was collected, and the heart, liver, spleen, lung, and kidney were harvested for H&E staining, blood routine tests and blood biochemical tests were performed to comprehensively evaluate the drug safety in mice during the treatment cycle.

*1.42. Statistical analysis.*

Quantitative data are presented as mean ± standard deviation (SD). Statistical analyses were performed using GraphPad Prism 8.0. Comparisons among multiple groups at a single endpoint were performed using one-way ANOVA followed by Tukey’s multiple-comparison test. Dunnett’s multiple-comparison test was used when multiple treatment concentrations were compared with a single control group. Longitudinal tumor volume, body weight, and bioluminescence data were analyzed using two-way repeated-measures ANOVA followed by Šídák’s multiple-comparison test. Survival curves were compared using the log-rank (Mantel-Cox) test. Sample sizes are specified in the corresponding figure legends. ns, not significant; **p* < 0.05, ***p* < 0.01, ****p* < 0.001 and *****p* < 0.0001.

**References**

[1] G. Kresse, J. Furthmüller, "Efficiency of ab-initio total energy calculations for metals and semiconductors using a plane-wave basis set," *Computational Materials Science* 6, no. 1 (1996), 15–50.
[2] J. P. Perdew, K. Burke, M. Ernzerhof, "Generalized Gradient Approximation Made Simple," *Physical Review Letters* 77, no. 18 (1996), 3865–3868.
[3] G. Kresse, D. Joubert, "From ultrasoft pseudopotentials to the projector augmented-wave method," *Physical Review B* 59, no. 3 (1999), 1758–1775.
[4] P. E. Blöchl, "Projector augmented-wave method," *Physical Review B* 50, no. 24 (1994), 17953–17979.
[5] H. J. Monkhorst, J. D. Pack, "Special points for Brillouin-zone integrations," *Physical Review B* 13, no. 12 (1976), 5188–5192.
[6] D. Hobbs, G. Kresse, J. Hafner, "Fully unconstrained noncollinear magnetism within the projector augmented-wave method," *Physical Review B* 62, no. 17 (2000), 11556–11570.
[7] A. A. Mostofi, J. R. Yates, G. Pizzi, et al., "An updated version of wannier90: A tool for obtaining maximally-localised Wannier functions," *Computer Physics Communications* 185, no. 8 (2014), 2309–2310.
[8] Q. Wu, S. Zhang, H.-F. Song, et al., "WannierTools: An open-source software package for novel topological materials," *Computer Physics Communications* 224 (2018), 405–416.
[9] S. Grimme, J. Antony, S. Ehrlich, H. Krieg, "A consistent and accurate ab initio parametrization of density functional dispersion correction DFT-D for the 94 elements H-Pu," The *Journal of Chemical Physics* 132, no. 15 (2010), 154104.
[10] J. K. Nørskov, J. Rossmeisl, A. Logadottir, et al., "Origin of the overpotential for oxygen reduction at a fuel-cell cathode," *The Journal of Physical Chemistry* 108, no. 46 (2004), 17886–17892.
[11] G. Du, L. B. Healy, L. David, et al., "ROS-dependent S-palmitoylation activates cleaved and intact gasdermin D," *Nature* 630, no. 8016 (2024), 437–446.

**2.** **Supplementary figures**

**Figure S1. Characterization of the Pd_3_Sn TQMs.** (a-c) Tauc plots derived from solid-state UV-vis diffuse reflectance spectra of Pd_3_Sn-Dots, Pd_3_Sn-Strip and Pd_3_Sn-Network. (d) Bulk band structure of Pd_3_Sn with spin-orbit coupling (SOC) along high-symmetry paths. (e) Total and projected density of states (DOS/PDOS) of Pd_3_Sn calculated with SOC, showing the orbital-resolved contributions (Pd(d), Sn(s) and Sn(p)). (f) Wannier-interpolated non-SOC nodal crossing between bands 13 and 14 along the M–Γ path. A closed loop enclosing the crossing yields a Berry phase of 0.9987π. (g) Calculated [111] Zak-phase map for the non-SOC Wannier Hamiltonian. (h) Calculated semi-infinite surface spectral functions of the Pd_3_Sn (111) and (100) terminations, showing facet-dependent surface-state dispersions.

**Figure S2. Electrochemical and catalytic performance of Pd_3_Sn nanostructures.** (a) LSV curves of Pd_3_Sn-Dots (D), Pd_3_Sn-Strip (S), and Pd_3_Sn-Network (N) measured in 0.1 M acetate buffer at pH 5.5. (b) LSV curves of the same samples in acetate buffer with 5 mM H_2_O_2_. (c-e) UV-vis spectra of TMB oxidation catalyzed by Pd_3_Sn-Dots (D) (c), Pd_3_Sn-Strip (S) (d) and Pd_3_Sn-Network (N) (e) under different conditions, confirming their OXD-like and POD-like activity. (f) US amplification index (Δk) of the three Pd_3_Sn morphologies extracted from DPBF degradation kinetics with and without US. (g-i) POD-like activity of Pd_3_Sn-Strip toward TMB/H_2_O_2_ under varied experimental parameters: (g) different Pd_3_Sn-Strip concentrations, (h) different H_2_O_2_ concentrations, and (i) different TMB concentrations. (j) CAT-like activity of Pd_3_Sn-Strip evaluated by time-dependent dissolved O_2_ release at various H_2_O_2_ concentrations. (k) Comparison of DPBF degradation by Pd_3_Sn-Strip and PS-bio. (l) Comparison of TMB oxidation catalyzed by Pd_3_Sn-Strip and PS-bio.

**Figure S3. Characterization of Pd_3_Sn-bio (PS-bio).** (a) Schematic illustration of the preparation of Pd_3_Sn-bio. (b) SAED pattern, dark-field TEM image and elemental mapping (O, Pd, Si, Sn) of Pd_3_Sn-bio. (c) DLS size distributions of Pd_3_Sn-Strip and Pd_3_Sn-bio in aqueous dispersion. (d) Zeta potentials of Pd_3_Sn-Strip and Pd_3_Sn-bio. (e) FTIR spectra of Pd_3_Sn, Pd_3_Sn@SiO_2_ and Pd_3_Sn@SiO_2_-bio (Pd_3_Sn-bio). (f) High-resolution XPS spectra of Sn 3d and Pd 3d regions. (g) XPS survey spectrum of Pd_3_Sn-bio.

**Figure S4. Colloidal stability of PS-bio.** (a) Hydrodynamic diameter and PDI of PS-bio dispersed in PBS at 37 ℃ over 48 h. (b) Hydrodynamic diameter and PDI of PS-bio dispersed in culture medium containing 10% fetal bovine serum at 37 ℃ over 48 h.

**Figure S5. US-activated PS-bio induces cytotoxicity and mitochondrial dysfunction *in vitro.*** (a, b) Cell viability was assessed by CCK-8 assay after 24-hour treatment with graded concentrations of PS-bio, with or without concurrent US (US, 1.0 W cm^-2^, 50% duty cycle, 2 min). (c) Live/dead staining using Calcein AM (green, live) and propidium iodide (PI, red, dead) for the indicated groups. The corresponding quantitative analysis of cell viability is presented in the bar graph. (d) Cytocompatibility of PS-bio in NCM460 cells assessed by CCK-8 assay (e) Mitochondrial membrane potential (ΔΨm) assessed by JC-1 staining. (f) Radial fluorescence intensity profiles of Rhodamine B labeled PS-bio from the spheroid surface toward the center after 1, 2, and 4 h incubation. Fluorescence intensity was normalized to the signal at the spheroid surface. Solid lines represent the mean and shaded regions represent SD from three independent spheroids. (g) Quantitative colocalization analysis of Rhodamine B-labeled PS-bio with Lyso-Tracker, expressed as Pearson's correlation coefficient (Pearson's r).

**Figure S6. GSDMD expression in human colorectal tumor tissues.** (a) Schematic workflow for collection and processing of tumor samples from patients. (b) Representative immunofluorescence images of GSDMD in normal colon and tumor tissues.

**Figure S7. Analysis of pyroptosis and inflammasome activation *in vitro*.** (a) Bright-field images of HT-29 cells after the indicated treatments (Control, US, PS-bio, PS-bio + US). (b) Quantification of Western blot band intensities of GSDMD-N, caspase-1, cleaved-caspase-1, and NLRP3 in the indicated groups (Control, US, PS-bio, PS-bio+US). (c) Intracellular ATP levels measured after the indicated treatments. (d) LDH release under the same conditions. (e) ELISA quantification of IL-1β and IL-18 secretion after treatment. Data are presented as mean ± SD, calculated using one-way ANOVA with **p* < 0.05, ***p* < 0.01, ****p* < 0.001, and *****p* < 0.0001.

**Figure S8. Acyl-biotin exchange (ABE) assay.** Schematic workflow of the ABE assay used to detect protein palmitoylation, including cell lysis, blocking of free thiols with NEM, HAM treatment or omission, labeling with HPDP-biotin, streptavidin-agarose enrichment, and subsequent Western blot analysis.

**Figure S9. Genetic validation of the role of zDHHC5 in GSDMD palmitoylation.** (a) Western blot analysis of zDHHC5 expression in HT-29 cells transfected with three independent ZDHHC5 targeting siRNAs (si-1, si-2, and si-3). β-actin was used as the loading control. (b) RT-qPCR analysis of relative ZDHHC5 mRNA expression following transfection with the indicated siRNAs. GAPDH was used as the internal reference gene. Data are presented as mean ± SD from three independent experiments. (c) ABE assay of GSDMD palmitoylation in the presence or absence of hydroxylamine (HAM), PS-bio + US treatment, and ZDHHC5 siRNA. Statistical analysis in (b) was performed using one way ANOVA followed by Dunnett’s multiple comparison test against the Control group.

**Figure S10. Conditioned medium from PS-bio + US treated cells promotes senescence in HT-29 cells.** (a) Representative SA-β-gal staining images of untreated HT-29 recipient cells cultured with conditioned medium collected from Control or PS-bio+ US treated donor cells. (b) Quantification of SA-β-gal positive recipient cells. Data are presented as mean ± SD.

**Figure S11. Flow cytometric assay.** Flow cytometric analysis of cell-cycle distribution in Control, US, PS-bio, PS-bio + US, and PS-bio + US + PL groups.

**Figure S12. Antitumor effect of PS-bio based treatment in a subcutaneous CT26 tumor model.** (a) Mean tumor volume versus time for the six groups. G1: Control; G2: US; G3: PS-bio; G4: PL; G5: PS-bio + US; G6: PS-bio + US + PL (n = 5 per group). (b) Body weight of mice recorded during the treatment period. (c) Representative Ki-67 immunohistochemical staining of tumor sections from the indicated groups. (d) Representative H&E staining of tumor sections from the indicated groups. Scale bars are indicated in the images. (e) Western blot analysis of full length GSDMD and the cleaved GSDMD-N in tumor tissues from the Control, US, PS-bio, and PS-bio + US groups. (f) Densitometric quantification of GSDMD-N expression normalized to Vinculin. Data in (a) and (b) are presented as mean ± SD (n = 5). Tumor-volume and body-weight curves were analyzed using two-way repeated-measures ANOVA followed by Šídák’s multiple-comparison test. Data in (f) are presented as mean ± SD (n = 3) and were analyzed using one-way ANOVA followed by Tukey’s multiple comparison test.

**Figure S13. Histological and hemocompatibility evaluation of PS-bio in tumor-bearing mice.**
(a) Representative H&E staining of major organs (heart, liver, spleen, lung, and kidney) collected from mice in different treatment groups (Control, US, PS-bio, PL, PS-bio + US, and PS-bio + US + PL). (b) *In vitro* hemolysis assay (n = 3).

**Figure S14. Hematology and serum biochemistry analysis after systemic treatment.**
(a) Complete blood count parameters of mice from each treatment group. (b) Serum biochemistry parameters for the same groups. Data are presented as mean ± SD with individual data points shown (n = 3 mice per group). Statistical comparisons were performed using one-way ANOVA followed by Tukey’s multiple comparison test, and no significant differences were detected among the treatment groups *(p > 0.05).*
